# An ICP-MS study on metal content in biodiesel and bioglycerol produced from heated and unheated canola oils

**DOI:** 10.1007/s11356-023-30004-x

**Published:** 2023-10-25

**Authors:** Rukayat S. Bojesomo, Abhijeet Raj, Mirella Elkadi, Mohamed I. Hassan Ali, Sasi Stephen

**Affiliations:** 1 Department of Chemistry, Khalifa University of Science and Technology, P.O Box: 127788, Abu Dhabi, United Arab Emirates; 2 Department of Chemical Engineering, Indian Institute of Technology Delhi, New Delhi, 110016 India; 3 Department of Mechanical Engineering, Khalifa University of Science and Technology, P.O Box: 127788, Abu Dhabi, United Arab Emirates

**Keywords:** Waste cooking oil, Canola biodiesel, Trace metals, Thermal effect, Response surface methodology, FTIR, Elemental analysis

## Abstract

This study addresses the challenges of biodiesel production costs and waste oil disposal by investigating the use of low-cost waste oil as a feedstock. The impact of heating temperature on biodiesel yield and trace metal levels is examined using response surface methodology (RSM). Optimal conditions for high biodiesel yields (95–98%) from canola oil are determined with a methanol/oil ratio of 12:1, 1 wt% catalyst, and 60-min reaction time. For crude bioglycerol, the optimal conditions involve a methanol/oil ratio of 4.25:1, 2.93 wt% catalyst, and 119.15-min reaction time. Elemental analysis reveals the presence of high-concentration metals like Cu and Zn and low-concentration ones such as Pb, As, Se, and Zr in both oil feedstocks and their respective biodiesel and bioglycerol products. The study demonstrates that thermal stress on canola oil significantly impacts biodiesel and bioglycerol yields and trace metal levels during the transesterification process. The findings contribute to enhancing cost-effectiveness and environmental sustainability in biodiesel production.

**Supplementary Information:**

The online version contains supplementary material available at 10.1007/s11356-023-30004-x.

## Introduction

In recent decades, the increasing global energy demand, the concerns over fossil fuel depletion, and the escalating pollution levels have sparked a growing interest in eco-friendly and sustainable fuels like biodiesel and bioethanol (Ambaye et al. 2021; Demirbas 2008, 2009). Biodiesel is a monoalkyl ester of long-chain unsaturated fats derived from edible and inedible vegetable oils or animal fats, serving as an alternative to petrodiesel (Akram et al. 2022; Bayraktar et al. 2023; Patel and Brahmbhatt 2022). The transesterification process is employed to produce biodiesel and bioglycerol from oils in a 3:1 ratio by mole (Ambaye et al. 2021). To accelerate this conversion, a basic, acidic, or biological catalyst is needed (Changmai et al. 2020; Mandari and Devarai 2022; Manojkumar et al. 2022; Zahed et al. 2018). The successful transesterification of oil depends on many factors such as the nature of the oil feedstock and the presence of free fatty acids (FFA) in it. However, the high cost associated with the refined oil feedstock poses a significant challenge to biodiesel commercialization. To address this, utilizing inexpensive and readily available oil feedstocks such as waste cooking oil (WCO) or used cooking oil (UCO), can effectively reduce production costs and contribute to environmental waste management (Mathew et al. 2021; Singh et al. 2021). In addition, heating vegetable oils at high temperatures degrades their physicochemical properties for both culinary and biofuel applications. Frying is one of the most common cooking methods, and a considerable portion of oil used for frying is disposed of after use, which causes drainage issues, degradation of the quality of wastewater, and the loss of a commodity that could produce biodiesel (Giuffrè et al. 2017). The concentration of FFA in triglycerides increases with increased heating or frying temperature, and biodiesel yield decreases owing to soap (saponification) and water production. To mitigate these side reactions and maximize the yield of biodiesel, some studies have used a two-step method: acid pretreatment followed by a base-catalyzed transesterification reaction (Patil and Deng 2009; Sahar et al. 2018) and the use of co-solvents such as tetrahydrofuran (THF) (Lam and Lee 2010; Roschat et al. 2016). To optimize biodiesel yield, several studies (Corral Bobadilla et al. 2017; Hamze et al. 2015) have used response surface methodology (RSM) to investigate and establish the correlation among the reaction parameters to maximize biodiesel generated from waste cooking oil. These studies, however, are confined to certain oils and a limited thermal stress to which oil has been subjected to. The low-cost biodiesel production from waste cooking oil for commercialization will include collecting it from various sources regardless of the heating temperature. Finding an optimal condition, irrespective of heating temperature, for enhancing the yield of biodiesel from waste cooking oil is very crucial in this case. RSM is a statistical approach for designing, optimizing, and analyzing trials in any process. The traditional one-variable-at-a-time optimization method cannot offer information regarding the interaction and quadratic impacts of process variables. The central composite design (CCD) and the Box-Behnken design (BBD) are the two RSM methods used to investigate process variables at five and three levels, respectively (Manojkumar et al. 2022).

The presence of heavy or trace metals in the oil feedstock can be transferred to biodiesel and bioglycerol during their production, even at low concentrations. This poses an environmental concern, and sensitive methods are necessary for accurate analysis due to the serious implications of heavy metal pollution. Ensuring the safety of biodiesel for human health and the environment requires elemental level analysis as a part of its quality control (Lepri et al. 2011; Roveda et al. 2018). Inductively coupled plasma mass spectrometry (ICP-MS) is a highly sensitive technique used for this purpose. It involves ionizing the sample with an inductively coupled plasma and then quantifying the elements present in it. Compared to atomic absorption spectroscopy (AAS), ICP-MS excels in terms of speed, accuracy, and sensitivity. This powerful method can detect metals and various non-metals in liquid samples at extremely low concentrations. Additionally, it can differentiate between different isotopes of the same element, making it valuable for isotopic labeling applications (Helaluddin et al. 2016; Korn et al. 2007; Todolí 2019; Woods and Fryer 2007). This technique is widely used for detecting metallic pollutants in complex environmental samples (Cregut et al. 2022) and the presence of trace metals on synthesized materials (Li et al. 2022; Wang et al. 2022). Although some studies have explored the elemental composition of biodiesel (Amais et al. 2012; de Souza et al. 2011; Elkadi et al. 2014; Ghisi et al. 2011; Lepri et al. 2011; Pereira et al. 2014; Pillay et al. 2012; Sánchez et al. 2015, 2014; Woods and Fryer 2007), very few studies have looked at the elemental composition of biodiesels and their oil feedstock together (Chaves et al. 2011; Lepri et al. 2011), and no study has reported the effect of thermal stress on the distribution of metallic and non-metallic elements from oil to biodiesel and biogylcerol phases produced from it.

The conversion of bioglycerol to value-added products has been one of the most significant topics of contemporary sustainability research (Devi and Dalai 2020). The presence of metal impurities in bioglycerol will have a detrimental impact on its exploitation and will require its pretreatment for metal removal to increase its purity.

It is clear from the above discussion that the studies present in the literature have not yet looked at the variations in the yields of biodiesel and bioglycerol that can arise during the transesterification process with the changes in the temperature at which oil was heated while cooking or frying, especially when the oil is heated near its smoke point. Moreover, it is also not clear from the studies reported so far on how the amount of metals present in oil varies with its heating temperature and how much of them can get transferred to biodiesel and bioglycerol that were produced from oil through transesterification. It is important to understand this given the negative impact of trace metals in fuels on human health and environmental pollution. In these directions, this study aims to experimentally explore the effect of heating an oil at different temperatures (below and near the smoke point) on the optimum operating parameters (methanol-to-oil ratio, catalyst concentration, and reaction time) for its conversion to biodiesel and bioglycerol through transesterification. The elemental analyses of the heated and unheated oils as well as the biodiesels and bioglycerols will be carried out through ICP-MS to track the transfer of trace metals from oil feedstock to the final products and determine if their concentrations are below the acceptable limits. Canola oil, often known as rapeseed oil, has been chosen for this study, as its biodiesel comfortably meets the international biodiesel requirements (known as EN-14214 in Europe and ASTM D 6751 in the United States) (Devi and Dalai 2020). To determine the optimum conditions for the single-step base-catalyzed transesterification of thermally stressed canola oil to biodiesel and bioglycerol, the RSM statistics to design the experiments and the conventional single-factor optimization methods will be employed. The effects of oil heating temperature on the elemental composition of biodiesel and bioglycerol will also be discussed.

## Materials and methods

### Materials

Potassium hydroxide, nitric acid, and analytical grade methanol were purchased from Sigma-Aldrich and were used without further purification. Refined canola oil was purchased from the local store in Abu Dhabi, UAE.

### Sample preparation

Before the transesterification step of the unheated oil, the sample of the refined canola oil was dried in an oven at 100 °C (denoted as CO_100) for 1 h to remove the moisture (Banerjee and Chakraborty 2009; Demirbas 2009) since the presence of water in oil negatively impacts the yield of methyl esters in catalytic conversion methods (Kusdiana and Saka 2004) and the hydrolysis of triglycerides by water can lead to soap formation during transesterification process (Moazeni et al. 2019). Heated oil was produced by placing 500 mL of canola oil in a 1-L beaker and heating it to either 190 °C (labeled CO_190) or 240 °C (labeled CO_240). These two temperatures were chosen based on the frying temperature (190 °C) and the temperature that can be reached during deep-frying (240 °C), which is also close to the canola oil smoke point (Fullana et al. 2004; Saleem and Ahmad 2018; Yao et al. 2015). The samples were subjected to each temperature for a duration of 120 min. Subsequently, the heated oil was allowed to cool down to room temperature and analyzed within a period of 2 h.

### Transesterification process

In this study, the process of producing biodiesel and bioglycerol involved subjecting the prepared oil samples to a transesterification reaction using a homogenous catalyst (KOH) and methanol. A homogenous catalyst was chosen for transesterification due to its simplicity, shorter reaction duration, and the ability to provide a high biodiesel yield (Devaraj et al. 2020; Rajendran et al. 2022). Although both acid and base catalysts can be utilized as homogeneous catalysts (Devaraj et al. 2020; Singh et al. 2023), alkali-based catalysts have been reported to be significantly faster (up to four thousand times) as compared to acid catalysts (Sadia et al. 2018). Moreover, alkali catalysts are widely used in the industrial-scale production of biodiesel. The acid catalysts require more methanol to enhance biodiesel output. While they require low reaction temperatures for reaction, their corrosive nature poses operational challenges (Rajendran et al. 2022). Hence, an alkali catalyst (KOH) was selected and employed to ensure efficient biodiesel production.

Additionally, methanol was selected over ethanol in the transesterification process due to its advantages such as low price and high biodiesel yield despite its toxicity. Moreover, the synthesis of fatty acid ethyl esters (FAEE) poses more challenges as compared to fatty acid methyl esters (FAME), particularly during purification, as FAEE, ethanol, and glycerol form a difficult-to-separate tri-phasic system. Achieving the required ester content in biodiesel is relatively simpler with FAME, but is not as easily achieved with FAEE (Musa 2016; Ortega et al. 2021; Sanli and Canakci 2008).

In the experiments, an appropriate weight of the catalyst (KOH) was dissolved (amounts varying from one experiment to another) in a given volume of methanol. Half of the volume of methanol/catalyst mixture was transferred into a pre-weighed oil sample inside a two-neck round-bottom flask and stirred continuously for 10 min at room temperature. The temperature was then raised to 50 °C, and the remaining catalyst/methanol mixture was added. The stirring/reflux was continued for 60 min (depending on the trial design) at 55 ± 5 °C. The product of transesterification, which formed a biphasic mixture, was carefully transferred into a separating funnel and subsequently separated into its individual phases. The lower layer (bioglycerol) was dried in the oven at 100 °C to evaporate residual methanol and water content, while the upper layer (biodiesel) was washed to remove the residual catalyst and methanol several times until the water became clear and was then dried in the oven at 100 °C. The percentage yield of biodiesel or crude bioglycerol was calculated by taking the ratio of the weight of biodiesel or crude bioglycerol to the weight of oil and multiplying by hundred. Each experiment was conducted three times (triplicate), and the average of the numerical data obtained from these replicates is presented in the results.

### Design of experiments and statistical analysis

Response surface methodology (RSM) was employed to optimize the biodiesel production process using heated canola oil. The study also investigated the influence of various transesterification process variables on the yields of both biodiesel and bioglycerol. The experimental design utilized the central composite design (CCD) method to comprehensively explore the effects of these variables. The independent variables (Table 1) were the methanol-to-oil molar (M/O) ratio (A), catalyst wt% (B), and the reaction duration (C), whereas the dependent variables were the biodiesel and crude bioglycerol yields (response). The three-factor (A, B, C) test matrix is repeated three times at three temperature levels. Therefore, T is not included in the factual analysis, and thus, three Y models (one for each T) are obtained and analyzed. The actual yield was the ratio of the weight of biodiesel or crude bioglycerol to the weight of oil.

**Table 1:** Experimental ranges and levels of independent variables for the central composite design (CCD) method

Independent variable	Notation	Units	Ranges and levels				
			− *α*	− 1	0	+ 1	+ *α*
Methanol:oil molar ratio	A		3:1	6:1	9:1	12:1	15:1
Catalyst concentration	B	wt%	0.5	1.0	1.5	2.0	2.5
Reaction Time	C	min	30	60	80	100	120
Temperature	T	°C	100	190	240		

In total, twenty experimental runs were carried out at five levels of independent variable range (− *α*, − 1, 0, + 1, and + *α*) (as shown in Table 1), where each numeric factor is set to 5 levels: ± *α* (axial points), ± 1 (factorial points), and the center point. To discover the link between the independent variables and the responses, the obtained experimental data were analyzed using a second-order polynomial, as shown by Eq. 1 (Silva et al. 2011).

1 $$Y= {\beta }_{0}+ \sum_{j=1}^{k}{\beta }_{j}{x}_{j}+ \sum \sum_{i<j}{\beta }_{ij}{x}_{j}{x}_{j}+ \sum_{j=1}^{k}{\beta }_{jj}{x}_{j}^{2}+ \varepsilon$$

Here, *𝑌* is the response (% yield of biodiesel or bioglycerol); *β*_*0*_ is the intercept; *β*_*j*_, *β*_*jj*_, and *β*_*ij*_ are the linear, quadratic, and interactive coefficients, respectively; *k* is the number of factors; *x*_*i*_ and *x*_*j*_ are the independent variables under study (*x*_1,_ *x*_2_, and *x*_3_ represent A, B, and C, respectively, from Table 1); and ε is the residual error.

The statistical analysis of the model, including analysis of variance (ANOVA), was conducted to evaluate the experimental data. Design-Expert software was utilized to design the experiments and perform regression and graphical analysis for both response surface methodology (RSM) and single-factor optimization. The coded values of the independent variables for biodiesel and crude bioglycerol experiments can be found in Table 2.

**Table 2:** Full factorial central composite design matrix for biodiesel production

Run	Independent variables			Reponses (BD-100)		Reponses (BD-190)		Reponses (BD-240)	
	A: Methanol/oil ratio	B: Catalyst concentration (wt%)	C: Reaction time (min)	Actual yield (%)	Predicted yield (%)	Actual yield (%)	Predicted yield (%)	Actual yield (%)	Predicted yield (%)
1	− 1 (6.00)	− 1 (1.00)	− 1 (60.00)	89.53	88.98	91.3	87.68	98.04	92.17
2	+ 1 (12.00)	− 1 (1.00)	− 1(60.00)	96.47	98	96.7	98.24	98.73	96.93
3	− 1 (6.000)	+ 1 (2.00)	− 1 (60.00)	79.75	78.42	72.96	69.95	89.39	89.11
4	+ 1 (12.00)	+ 1 (2.00)	− 1 (60.00)	84.33	86.99	87.39	81.5	94.38	93.87
5	− 1 (6.00)	− 1 (1.00)	+ 1 (100.00)	94.91	92.82	86.52	87.75	79.06	88.81
6	+ 1 (12.00)	− 1 (1.00)	+ 1 (100.00)	94.83	96.73	92.67	91.01	97.41	93.57
7	− 1 (6.00)	+ 1 (2.00)	+ 1 (100.00)	76.14	75.18	81.63	75.42	87.41	85.75
8	+ 1 (12.00)	+ 1 (2.00)	+ 1 (100.00)	77.51	78.64	80.71	79.67	89.26	90.51
9	− *α* (3.00)	0 (1.50)	0 (80.00)	71.97	74.73	72.01	75.48	88.26	86.58
10	+ *α* (15.00)	0 (1.50)	0 (80.00)	90.53	87.21	89.09	90.29	94.35	96.1
11	0 (9.00)	− *α* (0.50)	0 (80.00)	96.29	96.28	95.45	93.22	96.47	94.4
12	0 (9.00)	+ *α* (3.00)	0 (80.00)	46.42	46.17	44.58	47.64	88.31	86.75
13	0 (9.00)	0 (1.50)	− *α* (30.00)	94.00	93.38	87.89	90.13	94.44	95.54
14	0 (9.00)	0 (1.50)	+ *α* (120.00)	88.93	89.32	85.93	87.09	93.6	87.98
15	0 (9.00)	0 (1.50)	0 (80.00)	93.94	93.38	84.55	86.07	94.62	91.34
16	0 (9.00)	0 (1.50)	0 (80.00)	92.22	93.38	83.69	86.07	83.21	91.34
17	0 (9.00)	0 (1.50)	0 (80.00)	94.31	93.38	85.6	86.07	92.25	91.34
18	0 (9.00)	0 (1.50)	0 (80.00)	93.1	93.38	83.92	86.07	91.67	91.34
19	0 (9.00)	0 (1.50)	0 (80.00)	93.92	93.38	84.25	86.07	89.45	91.34
20	0 (9.00)	0 (1.50)	0 (80.00)	89.53	88.98	84.67	86.07	85.79	91.34

### Elemental analysis (ICP-MS)

ICP-MS is one of the most accurate techniques for the quantitative elemental analysis of trace elements (Meermann and Nischwitz 2018; Moldovan et al. 2004). Other instruments that can aid in the elemental analyses are either less sensitive or have poor selectivity. For instance, in Olesik (1991), it was reported that the detection limit of ICP-MS was better than that of inductively coupled plasma optical emission spectroscopy (ICP-OES) by few orders of magnitude. In Trejos et al. (2013), the ICP methods were found to be better than X-ray fluorescence (XRF) methods in distinguishing samples with small differences in composition. In Pillay (2001) as well, ICP-MS has been reported to be more suitable for heavy element detection than XRF and particle induced X-ray emission (PIXE). The high sensitivity and selectivity of ICP-MS combined with less matrix interference and the unavailability of other sensitive instruments in our laboratory for trace metal analysis led to its choice for this study. PerkinElmer NexION 2000 instrument was used for the analysis of the samples. The instrument was used at an RF power of 1600 W. The plasma gas flow was set at 14.5 L/min, the auxiliary gas flow was at 1.2 L/min, and the nebulizer gas flow was at 0.94 L/min. The instrument was run in the kinetic energy discrimination (KED) mode to get rid of the unwanted polyatomic interferences from the actual elemental measurements. Helium was used as the collision cell gas and was fed at the rate of 5 mL/min. Approximately 500 mg of sample was digested in 5 mL of 70% ultra-pure HNO_3_ (Fluka Analytical). The digestion process was done using an industrial grade microwave oven (Anton Paar Multiwave PRO). A closed vessel system was used to digest the samples to minimize the sample loss. The samples were further diluted using ultra-pure water (Milli-Q Elemental, Merck Millipore) so that the final concentration of HNO_3_ was less than 5%. The nebulizer-spray chamber combination produced a fine aerosol of the sample, and a portion of the aerosol was transported into the hot plasma (the temperature of plasma usually ranges between 5000 and 7000 K). The ionized samples, after passing through the collimators, collision cell, and quadrupole mass selection system, reach the detector and are quantified. The electron multiplier tube detector operating in the dual mode (pulse stage voltage 950 V and analogue stage voltage − 1675 V) provides an amplified signal with wide dynamic range.

The instrument was calibrated using a standard calibration mixture, and the measurement accuracy was ensured by running a quality control standard. The expected value of each element was 10 ppb, and the reported mean values (in ppb) with their standard deviation are Ba 9.9 ± 3.2%, Be 9.8 ± 1.7%, Co 10.0 ± 2.5%, Cr 9.9 ± 2.9%, Cu 10.0 ± 2.0%, Mn 10.2 ± 2.8%, and V 9.9 ± 3.7%. All measurements were within a standard deviation of 5%.

## Results and discussion

### Optimization of transesterification reaction parameters for biodiesel production

The production of biodiesel was achieved through a one-step, homogenous catalyzed transesterification process. To optimize this process, independent variables, namely methanol/oil (M/O) ratio, catalyst wt%, and reaction time, were studied. Both conventional single-factor optimization and response surface methodology (RSM) with central composite design (CCD) were employed to analyze the interaction among these operational variables. This comprehensive approach aimed to identify the optimal conditions for biodiesel and bioglycerol production.

#### Statistical analysis

The biodiesel yields, both predicted and experimental, are presented in Table 2, following the procedure outlined in the “Design of experiments and statistical analysis” section. To assess the significance and suitability of the quadratic regression model, an analysis of variance (ANOVA) was conducted. Table 3 summarizes the ANOVA results for the full quadratic model, covering the percentage yield of biodiesel from unheated (CO_100) and heated canola oils (CO_190 and CO_240), denoted as BD_100, BD_190, and BD_240, respectively.

**Table 3:** ANOVA summary for the full quadratic model for percentage yield of biodiesel from unheated and heated canola oils

Source	BD_100							BD_190						BD_240					
	df	Sum of Squares	df	Mean Square	*F*-value	*p*-value		df	Sum of Squares	Mean Square	*F*-value	*p*-value		df	Sum of Squares	Mean Square	*F*-value	*p*-value	
Model	9	2730.27	9	303.36	68.74	< 0.0001	significant	9	2163.74	240.42	15.46	< 0.0001	significant	3	191.16	63.72	3.29	0.0478	significant
A-Methanol : Oil Molar Ratio	1	112.68	1	112.68	25.53	0.0005		1	180.47	180.47	11.61	0.0067		1	90.51	90.51	4.68	0.0461	
B–Catalyst Concentration	1	1634.50	1	1634.50	370.34	< 0.0001		1	1591.87	1591.87	102.39	< 0.0001		1	49.02	49.02	2.53	0.1311	
C-Reaction Time	1	36.91	1	36.91	8.36	0.0161		1	4.718E-06	4.718E-06	3.035E-07	0.9996		1	51.50	51.50	2.66	0.1224	
AB	1	0.1019	1	0.1019	0.0231	0.8822		1	0.4837	0.4837	0.0311	0.8635							
AC	1	13.08	1	13.08	2.96	0.1159		1	26.66	26.66	1.71	0.2197							
BC	1	25.05	1	25.05	5.68	0.0384		1	14.58	14.58	0.9378	0.3557							
A^2^	1	238.58	1	238.58	54.06	< 0.0001		1	15.75	15.75	1.01	0.3379							
B^2^	1	503.25	1	503.25	114.03	< 0.0001		1	210.27	210.27	13.52	0.0043							
C^2^	1	8.08	1	8.08	1.83	0.2058		1	8.92	8.92	0.5734	0.4664							
Residual	10	44.13	10	4.41				10	155.47	15.55				16	309.74	19.36			
Lack of Fit	5	41.10	5	8.22	13.55	0.0062	significant	5	153.19	30.64	66.97	0.0001	significant	11	217.87	19.81	1.08	0.5009	not significant
Pure Error	5	3.03	5	0.6069				5	2.29	0.4575				5	91.87	18.37			
Cor Total	19	2774.41	19					19	2319.21					19	500.90				

The model’s high *F*-value of 68.74 indicates its significance, with only a minute 0.01% possibility of such a large *F*-value occurring due to random variation (noise). Furthermore, a *P*-value below 0.05 confirms the importance of the model terms, namely *A*, *B*, *C*, *BC*, *A*^2^, and *B*^2^. The predicted *R*^2^ value of 0.8763 shows reasonable agreement with the adjusted *R*^2^ of 0.9698, with a difference below 0.2. Additionally, the precision of the model, with a signal-to-noise ratio of 34.889, indicates a strong signal and reliable performance. Consequently, this model is suitable for exploring the design space concerning BD_100.

The statistical analysis of biodiesel production from both unheated and heated canola oils was conducted. For BD_190, the *F*-value of 15.46 indicates the model’s significance, with a minimal 0.01% chance of such a large *F*-value occurring due to noise. However, there seems to be some discrepancy between the predicted *R*^2^ (0.1561) and the adjusted *R*^2^ (0.8726). In the case of BD_240, the *F*-value of 3.29 also suggests model suitability, with a 4.78% probability of such a large *F*-value occurring due to noise. The reported *P*-value below 0.05 further confirms the significance of the model terms. Moreover, the predicted *R*^2^ of 0.0684 reasonably agrees with the adjusted *R*^2^ of 0.2657 for BD_240. Thus, the reduced response model, represented by Eqs. 2, 3, and 4, effectively describes the percentage yield of biodiesel from both unheated and heated canola oils.

2 $${Y}_{\mathrm{BD}\_100}=89.56+6.77A-23.95B-4.02C-0.56AB-5.75AC-9.95BC-12.41{A}^{2}-17.86{B}^{2}-2.28{C}^{2}$$

3 $${Y}_{\mathrm{BD}\_190}=81.95+8.56A-23.6B-0.001C-1.23AB-8.21AC-7.59BC-3.19{A}^{2}-11.544{B}^{2}-2.40{C}^{2}$$

4 $${Y}_{\mathrm{BD}\_240}=90.99+4.76A-3.82B-3.78C$$

Here, *Y*_BD_100_, *Y*_BD_190_, and *Y*_BD_240_ are predicted percentage yields of biodiesel from unheated canola oil (dried at 100 °C), heated canola oil at 190 °C, and heated canola oil at 240 °C, respectively; *A* is M/O molar ratio, *B* is the catalyst wt% (wt%), and *C* is the reaction time (min). All the studied factors were found to significantly affect the percentage yield of biodiesel.

The significance of each parameter in influencing biodiesel yield was assessed using the probability value (*P*-value) listed in Table 3. A *P*-value of less than 0.005 indicates a significant effect of the parameters at a 95% confidence level. The results of the variance analysis (ANOVA) in Table 3 reveal that the catalyst wt% and M/O ratio hold the most significant influence on biodiesel yield from both heated (BD_190 and BD_240) and unheated canola oils (BD_100). This is supported by their low *P*-values and high *F*-values, which is consistent with the findings in the existing literature (D’Cruz et al. 2007; Hamze et al. 2015; Zahed et al. 2018). An exception is BD_240, where the *P*-value is slightly higher and *F*-value is smaller for all parameters as compared to BD_100 and BD_190. While BD_100 and BD_190 adhere to the quadratic model, BD_240 aligns with a linear model, with a higher *R*^2^ value as compared to the quadratic model (Table S1 in the supplementary material). However, the reaction time proves to be a less significant factor in biodiesel production from both heated and unheated canola oils. Overall, the model is satisfactory for exploring the experimental relationship between variables and the response (yield) within the range of experimental variables considered in this study.

#### Diagnostic test (affirming the numerical model)

To verify the accuracy of the model, validation was conducted using the residual table (Table 4). This table displays the residual values for both biodiesel and bioglycerol responses, as well as the leverages, which indicate the observations with significant influence on the regression model coefficients. From the results, it is evident that the developed model effectively describes the experimental range under investigation.

**Table 4:** Residual and leverage for biodiesel and bioglycerol production (response)

Run Order	BD_100		BD_190		BD_240		BG_100		BG_190		BG_240	
	Residual	Leverage	Residual	Leverage	Residual	Leverage	Residual	Leverage	Residual	Leverage	Residual	Leverage
1	0.5559	0.607	3.62	0.607	5.87	0.217	–0.8255	0.607	0.1166	0.607	0.0970	0.607
2	–1.53	0.607	–1.54	0.607	1.80	0.217	1.55	0.607	2.99	0.607	–0.5892	0.607
3	1.33	0.597	3.01	0.597	0.2816	0.208	–2.22	0.597	1.49	0.597	–0.2611	0.597
4	–2.66	0.597	5.89	0.597	0.5147	0.208	–1.03	0.597	–1.96	0.597	–0.3744	0.597
5	2.09	0.620	–1.23	0.620	–9.75	0.223	0.5882	0.620	1.94	0.620	0.4804	0.620
6	–1.90	0.620	1.66	0.620	3.84	0.223	1.78	0.620	–1.51	0.620	0.3672	0.620
7	0.9601	0.605	6.21	0.605	1.66	0.213	–1.99	0.605	–3.01	0.605	0.6952	0.605
8	–1.12	0.605	1.05	0.605	–1.25	0.213	0.3850	0.605	–0.1355	0.605	0.0090	0.605
9	–2.75	0.677	–3.47	0.677	1.68	0.300	2.00	0.677	–0.2764	0.677	–0.4528	0.677
10	3.32	0.677	–1.20	0.677	–1.75	0.300	–1.56	0.677	0.2953	0.677	0.3467	0.677
11	0.0138	0.586	2.23	0.586	2.07	0.251	–2.21	0.586	–2.14	0.586	–0.1284	0.586
12	0.2465	0.901	–3.06	0.901	1.56	0.465^(2)^	1.18	0.901	0.9597	0.901	0.0099	0.901
13	0.6161	0.823	–2.24	0.823	–1.10	0.386	0.7399	0.823	–0.9458	0.823	0.4386	0.823
14	–0.3942	0.625	–1.16	0.625	5.62	0.275	–0.7156	0.625	1.50	0.625	–0.7914	0.625
15	0.5550	0.142	–1.53	0.142	3.28	0.050	–0.4088	0.142	–1.23	0.142	–0.7136	0.142
16	–1.16	0.142	–2.38	0.142	–8.13	0.050	1.48	0.142	0.8123	0.142	–1.76	0.142
17	0.9340	0.142	–0.4686	0.142	0.9107	0.050	0.4762	0.142	0.8123	0.142	1.76	0.142
18	–0.2800	0.142	–2.15	0.142	0.3357	0.050	0.5832	0.142	–0.2947	0.142	0.7724	0.142
19	0.5400	0.142	–1.82	0.142	–1.89	0.050	–0.0758	0.142	0.1153	0.142	0.3704	0.142
20	0.6300	0.142	–1.40	0.142	–5.55	0.050	0.2772	0.142	0.4713	0.142	–0.2756	0.142

The supplementary material includes two plots: Figure S1 and Figure S2. Figure S1 displays a normal plot, revealing that the data points are scattered randomly on it. Notably, over 85% of the points align with the standard line, which confirms the model’s  usefulness for all responses. On the other hand, Figure S2 illustrates the residual plot against the run order, and the random pattern of the residuals indicates the accuracy of the model. Both plots provide further validation for the reliability and effectiveness of the model.

#### Interaction effects of the independent variables

**Fig. 1:**
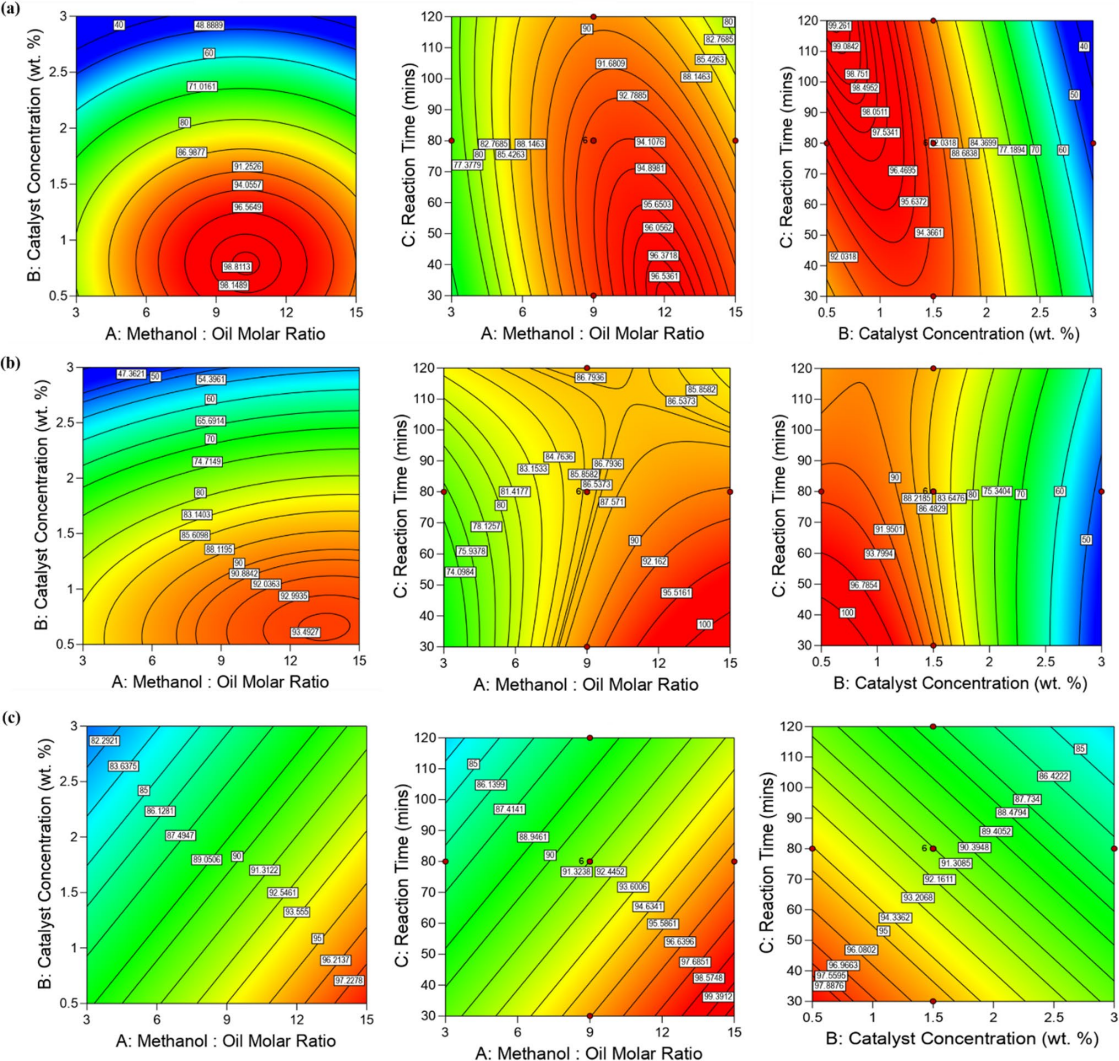
Contour plots showing the effects of methanol/oil ratio and catalyst wt% (left), methanol/oil ratio and reaction time (middle), and catalyst wt% and reaction time (right) on biodiesel yields from **a** unheated oil (BD_100); **b** heated oil at 190 °C (BD_190); and **c** heated canola oil at 240 °C (BD_240)

Figure 1 illustrates the contour plots of biodiesel yield (percent) for both heated and unheated canola oils. Due to the interaction effects among the variables, it is not possible to evaluate each parameter for BD_100 and BD_190 individually. The contour plots in Fig. 1 demonstrate the combined effects of three independent variables in a regression model equation. Each contour plot represents two independent variables (A, B, or C), while the third variable is held constant at a medium value (M/O ratio of 9:1, catalyst wt% of 1.5%, and reaction time of 90 min). These plots provide insights into the dependence and variation in response values when experimental conditions are altered.

For BD_100 and BD_190, all the parameter interactions, M/O ratio-catalyst wt% (MC), M/O ratio-reaction time (MR), and catalyst wt%-reaction time (CR), are considered significant. However, the interaction of MR is comparatively weaker when compared to MC and CR. Notably, Fig. 1c indicates that there is no interaction among the variables for BD_240. This finding aligns with the statistical analysis, which shows that the linear model exhibits a better fit with the highest *R*^2^ value (0.0684) and the lowest *P*-value (0.0478) as compared to the quadratic model (*R*^2^ =  − 0.4465 and *P*-value = 0.3522) in Table S1 of the supplementary material.

#### Effects of the main operating parameters on biodiesel yield

**Fig. 2:**
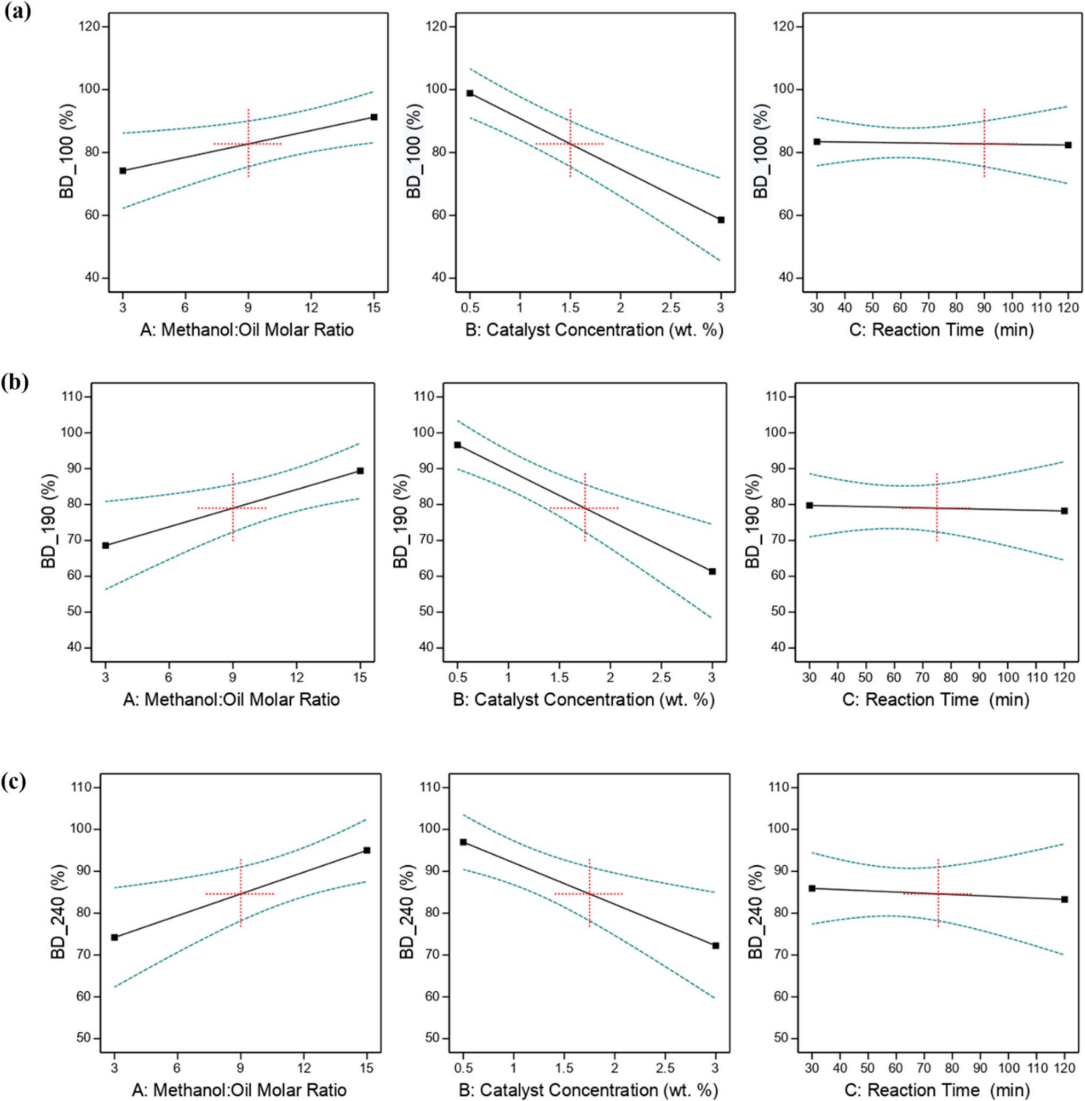
Conventional single factor plots on the effect of **a** methanol/oil ratio (left), catalyst wt% (middle), and reaction time (right) on the yield of biodiesel from **a** unheated canola oil (BD_100); **b** heated canola oil at 190 °C (BD_190); and **c** heated canola oil at 240 °C (BD_240)

Figure 1 a–c illustrate the contour plots, presenting the effects of independent variables during the RSM analysis. On the other hand, Fig. 2a–c showcase the outcomes of the conventional single-factor optimization analysis, which will be further discussed in the following subsections. To maintain the efficiency of the transesterification processes, temperatures were kept below methanol’s boiling point (64.7 °C), thereby minimizing methanol evaporation. This precautionary measure ensures that the reaction proceeds smoothly and reduces the likelihood of alkali catalyst-induced saponification before the reaction completion (Hamze et al. 2015; Harabi et al. 2019; Patil and Deng 2009).

##### Effect of M/O ratio

The interaction plots in Fig. 1 and the statistical analysis reveal that the methanol/oil ratio (A) plays a crucial role in affecting biodiesel yield. In the transesterification process, a molar ratio of 3:1 to 15:1 of methanol to canola oil was employed, as this ratio is stoichiometrically required to yield three moles of methyl ester (biodiesel) and one mole of bioglycerol (crude) (Manojkumar et al. 2022; Zahed et al. 2018). Both RSM analysis (Fig. 1) and single factor optimization (Fig. 2) indicate that increasing the M/O ratio from 3:1 to 12:1, along with the catalyst wt% from 0.5 to 1 wt%, leads to higher biodiesel yield. However, when pushing the M/O ratio beyond 12:1, biodiesel yield decreases significantly from 96 to 80%. This decline is attributed to the high solubility of bioglycerol in methanol, which hinders effective separation of bioglycerol from biodiesel and encourages the conversion of diglyceride to monoglyceride. As a result, the possibility of the combination of bioglycerol with monoglyceride arises via a reverse transesterification reaction.

##### Effects of catalyst concentration

The impact of catalyst wt% was investigated by varying it in the range of 0.5 to 3% for both the RSM and conventional single-factor optimization analyses. Figure 1 and Fig. 2 showcase the effect of catalyst amount, specifically in relation to M/O ratio and reaction time (Fig. 1) for the RSM analysis and M/O ratio (Fig. 2) for the single-factor analysis. Notably, an incremental rise in biodiesel yield was observed across all oil feedstocks considered in this study as the catalyst wt% increased from 0.5 to 1.0%. However, the biodiesel yield decreased when the catalyst wt% exceeded 1.0%. The optimal biodiesel yield, ranging from 95 to 98%, was achieved with a catalyst loading of 0.5 to 1.0% in both the RSM and single-factor analyses for all the studied oils (Figs. 1 and 2). These results align with the findings reported by Harabi et al. (2019), where a 0.5% catalyst loading yielded 96% biodiesel from waste frying oil. The reduction in biodiesel yield at high catalyst loadings (above 1%) can be attributed to soap formation resulting from the saponification reaction. Consequently, ester and glycerol emulsification occur, making it challenging to separate the biodiesel layer from bioglycerol. This leads to a decrease in biodiesel yield (Atapour et al. 2014; Harabi et al. 2019).

##### Effects of reaction time

To ensure optimal interaction between reactants during transesterification, continuous stirring was maintained throughout the reactions. The reaction time was varied between 30 and 120 min, but the ANOVA results suggest that it is a less significant variable compared to the other two independent variables, as evident from its high *P*-value and low *F*-value in the RSM analysis.

Both the contour plots obtained from the RSM analysis (Fig. 1) and the single-factor plots (Fig. 2) show subtle effects of reaction time on biodiesel yield for unheated and heated canola oils. A slight increase in the biodiesel yield was observed with increasing reaction time from 30 to 60 min in the single factor analysis. A similar trend was noticed in the RSM analysis with increasing M/O ratio and catalyst wt% (up to 1.25 wt%). A maximum yield of biodiesel was achieved at a reaction time of 60 min in both the RSM and single-factor analyses, which falls in line with the results reported by Patil and Deng (2009).

Beyond 60 min, the reaction time generally did not significantly influence biodiesel yield, though occasionally, slight reduction in the yield is observed in Fig. 2. This trend is consistent with the findings reported in Nadeem et al. (2021) for biodiesel from waste cooking oil, where methyl ester yield increased up to a certain point with rising reaction time and then stabilized. Similarly, Yuan et al. observed a slight decrease in biodiesel yield above 60-min reaction time for waste rapeseed oil with high FFA (Yuan et al. 2008).

### Optimization of transesterification reaction parameters

The optimal transesterification reaction conditions were determined using a numerical optimization tool within the DesignExpert software. Interestingly, the same values of M/O ratio (12:1), catalyst wt% (1.0 wt%), and reaction time (60 min) were found to yield maximum biodiesel from both heated and unheated canola oils in the RSM analysis, which is in accordance with the results obtained from the conventional single factor optimization analysis. This indicates that, even with severe heating up to 240 °C, the optimum process parameters for biodiesel production remained unchanged. However, the product yields were affected, as elaborated below.

**Fig. 3:**
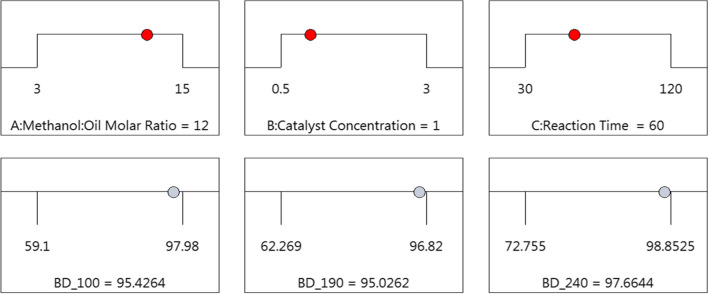
Optimum conditions obtained by RSM analysis to produce biodiesel from heated and unheated canola oils

The predicted yield was within the range of 95 to 98% with a desirability value of 1.0 (Fig. 3; Table 5). To verify the accuracy of the fitted model (Eqs. 2, 3, and 4), the predicted conditions were compared to those obtained in a similar single factor analysis (Figure S3 in the supplementary material), where a yield of 95 to 99% was reported for both unheated and heated canola oils, which aligned closely with the predicted values.

**Table 5:** Numerical optimization report for biodiesel

Number	Methanol/oil ratio	Catalyst wt%	Reaction time, min	BD_100	BD_190	BD_240	Desirability	Decision
1	*12*	*1*	*60*	95.426	95.026	97.664	1	Selected
2	*12.368*	*1.719*	*43.082*	93.857	90.927	96.442	1	
3	*10.849*	*0.896*	*109.798*	98.023	90.245	92.149	1	
4	*11.274*	*1.144*	*67.482*	98.022	94.458	95.284	1	
5	*11.169*	*1.383*	*37.489*	96.995	96.436	96.988	1	

A similar approach, as described earlier, was used to investigate the impact of reaction parameters on bioglycerol yield. Since the results align with those obtained for biodiesel production, a detailed discussion on bioglycerol yield is provided in the supplementary material (Figure S5 to Figure S8). Here, we present only the optimum conditions for achieving the maximum bioglycerol yield from both heated and unheated canola oils, which are M/O ratio of 4.25:1, a catalyst wt% of 2.93 wt%, and a reaction time of 119.15 min (Table 6). The predicted crude bioglycerol yield ranged between 25 and 60% based on the oil conditions considered in this study, as determined by the RSM analysis.

**Table 6:** Numerical optimization report for bioglycerol production

Number	Methanol/oil ratio	Catalyst wt%	Reaction time, min	BG_100	BG_190	BG_240	Desirability	Decision
1	*4.248*	*2.937*	*119.146*	60.543	57.13	24.918	1	Selected
2	*13.709*	*2.819*	*111.639*	43.966	39.784	23.903	1	
3	*3.9*	*2.952*	*65.801*	49.362	65.134	19.509	1	
4	*4.444*	*2.934*	*67.972*	47.721	61.306	19.477	1	
5	*14.818*	*2.935*	*75.348*	42.068	40.69	24.425	1	

The achieved optimum condition for the maximum yield is consistent with the findings of Kongjao et al. (2010) and Harabi et al. (2019). Similar to Harabi et al., the crude glycerol exhibited a dark brown color (Figure S4 in the supplementary material), indicating a high glycerol content, and has a pH of approximately 10 due to the residual KOH catalyst used in the transesterification reaction. The optimization of bioglycerol production is significant, as bioglycerol serves as a crucial precursor for the synthesis of nanomaterials like carbon nanotubes and carbon quantum dots that find applications in several fields such as hydrogen production, fuel cells, and chemical sensing (Devi and Dalai 2020).

## FT-IR analysis of biodiesel and bioglycerol

**Fig. 4:**
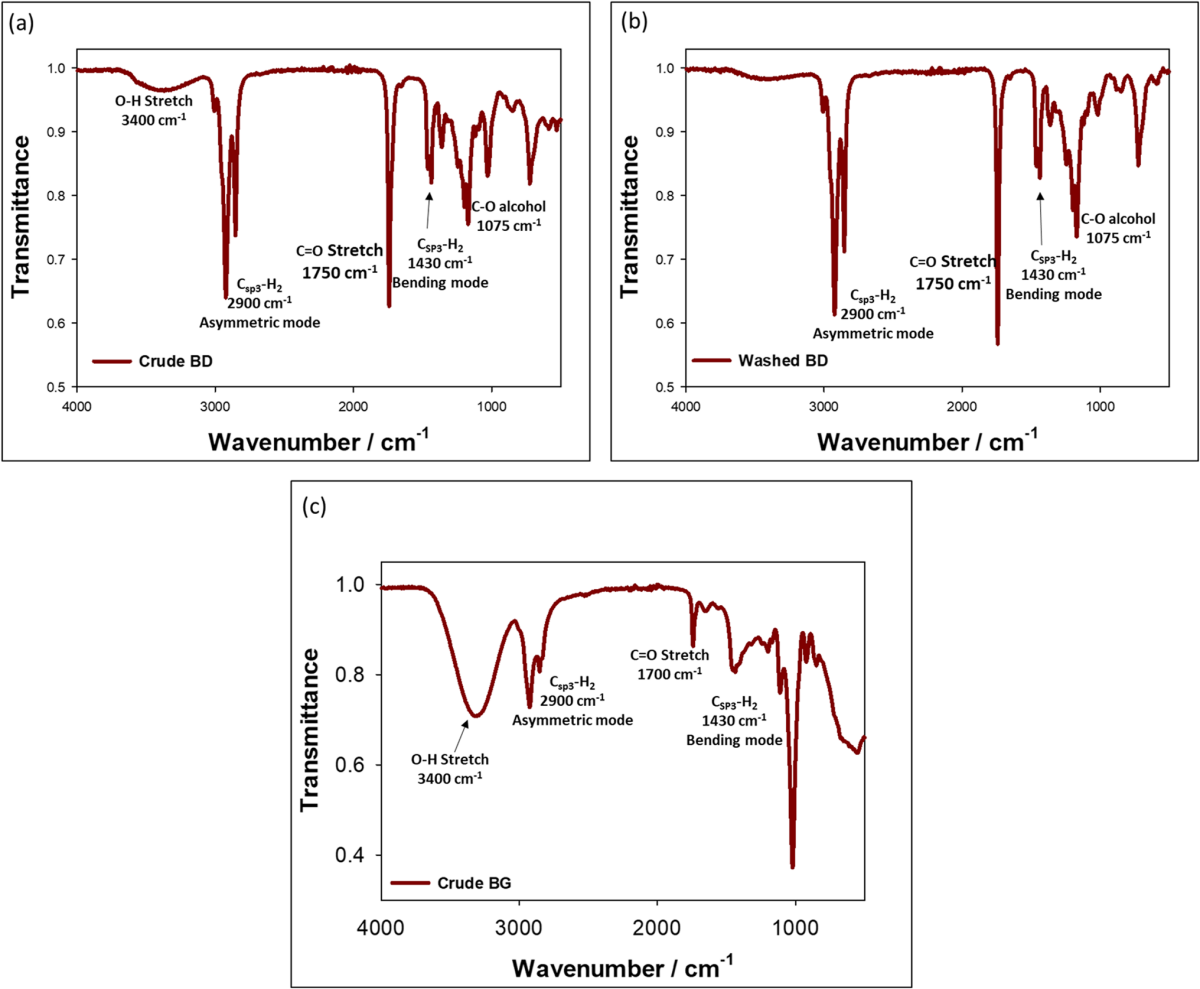
FT-IR spectra of **a** biodiesels after synthesis (crude BD), **b** after washing (washed BD), and **c** crude bioglycerol

Figure 4 illustrates the FT-IR spectra of biodiesel (before (Fig. 4a) and after washing (Fig. 4b)) and crude bioglycerol (Fig. 4c) obtained from heated canola oils. Notably, a significant peak at approximately 1750 cm^−1^ in the biodiesel spectrum and 1700 cm^−1^ in the crude bioglycerol spectrum corresponds to the ester carbonyl bond. Furthermore, a peak at 1075 cm^−1^ indicates the presence of C-O stretching, which is evident in both biodiesel and crude bioglycerol. The FT-IR spectra also show an OH-stretch at 3400 cm^−1^ in the crude biodiesel, which is most likely attributed to the residual bioglycerol (that was removed by washing). Additionally, both biodiesel and bioglycerol exhibit asymmetric sp^3^ carbon peaks at approximately 2900 cm^−1^, while bending sp^3^ carbon peaks are observed around 1430 cm^−1^. The crude bioglycerol sample displays a broad OH-stretch occurring near 3400 cm^−1^. These FT-IR analyses provide valuable insights into the chemical composition and functional groups present in biodiesel and crude bioglycerol.

### Physicochemical properties of unheated and heated canola oil biodiesel

Table 7 presents the physicochemical properties of unheated (BD_100) and heated (BD_190 and BD_240) biodiesels. The densities of BD_100, BD_190, and BD_240 fall within the range of US and EU biodiesel specifications, which is 860–900 kg/m^3^ as per EN ISO 3104. Additionally, the kinematic viscosity of all the biodiesels complies with the ASTM-D6751 specification of 1.96–6 cSt. These results indicate that the biodiesels meet the required standards for density and viscosity, making them suitable for various applications and in accordance with regulatory guidelines. The table also provides the cetane index, initial and final boiling points of biodiesels together with the temperatures required to vaporize 10% (T10), 50% (T50), and 90% (T90) of the fuel. While the cetane index remained nearly constant for all the three biodiesels which indicates negligible change in the autoignition property of the fuels; the boiling points of BD_100 were relatively lower than those for BD_190 and BD_240. This indicates a relatively higher volatility of BD_100 as compared to other fuels.

**Table 7:** Fuel properties of heated and unheated canola oil biodiesel index (test temperature 40 °C)

Fuel property	Unit	BD_100	BD_190	BD_240
Density	kg/m^3^	864.9	885.5	880.3
Cetane index	N/A	59.8	58.6	58
Initial boiling point (IBP)	°C	198.8	208	222
T10	°C	195	215.1	231.8
T50	°C	305.7	333	343
T90	°C	380	373	384
Final boiling point (FBP)	°C	349.7	385.9	366.8
Kinematic viscosity	m^2^/s	0.315 × 10^−5^	0.395 × 10^−5^	0.419 × 10^−5^
Dynamic viscosity	kg/m/s	0.00275	0.00359	0.00365

### Elemental analysis

Vehicle exhaust contains a diverse range of gases and solid particles, including organic and inorganic compounds. The pollutants such as nitrogen oxides and trace metals can pose serious health risks to living beings. For instance, certain trace metals like iron, chromium, and copper can lead to the formation of reactive oxygen species, causing lung inflammation and cancer when inhaled (Coufalík et al. 2019). The addition of specific combinations of trace metals to diesel fuel can increase its toxicity, even though these metals may not be individually harmful (Hedfi et al. 2013). Notably, certain metals like chromium, arsenic, cadmium, nickel, and lead are classified as carcinogens (Abdulaziz et al. 2022; Agarwal et al. 2017). Given these health and environmental risks, trace metal analysis of biodiesel, as a cleaner alternative fuel, is crucial.

**Fig. 5:**
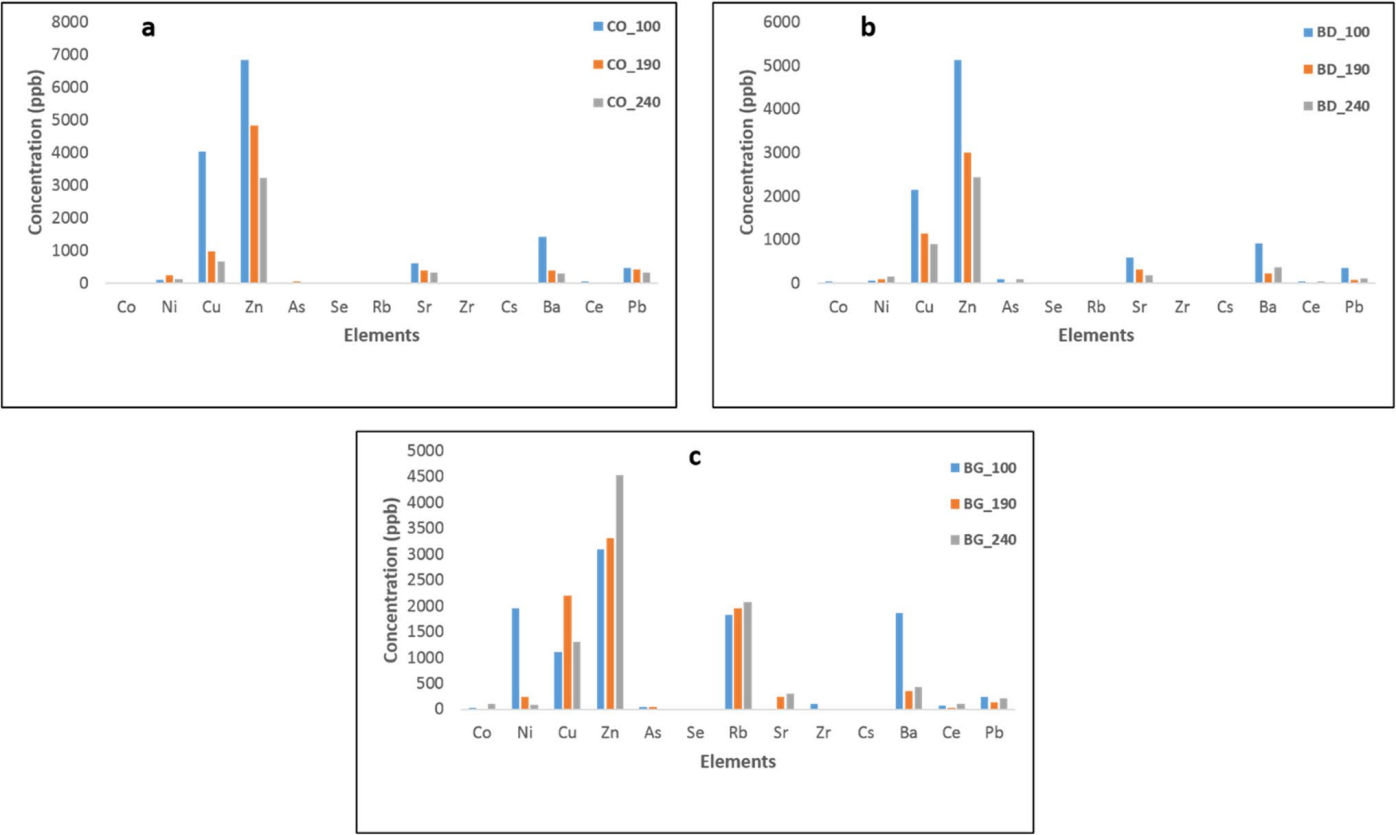
ICP-MS-detected elements and their concentrations in **a** canola oils, **b** biodiesels, and **c** crude bioglycerols (produced from oils heated at the temperatures of 100, 190, and 240 °C)

ICP mass spectrometry helps in determining the elemental composition of fuels and in identifying any potentially dangerous components. In the analysis, three categories of elements are formed based on their concentration levels: low (Pb, Ce, Co, As, Se, Zr, and S), mid (Rb, and Ni), and high (includes Zn and Cu), while Cs was not detected in any samples of canola oil, biodiesel, or crude bioglycerol. The concentration of elements generally decreased in canola oil as the heating temperature of the oil was increased, which indicates that some metals were lost during the oil heating process. For example, zinc, copper, and barium levels reduced from 6.8 ppm, 4.0 ppm, and 1.9 ppm in unheated canola oil to 2.4 ppm, 0.6 ppm, and 0.2 ppm, respectively, in canola oil heated at 240 °C for 2 h (Fig. 5). It is essential to analyze these metal concentrations in biodiesel to ensure its safety and prevent potential damage to diesel engines caused by high metal content in the fuel.

The investigation demonstrates that the trace metal concentrations in biodiesels derived from unheated (BD_100) and heated (BD_190 and BD_240) canola oils (Fig. 5b) exhibit a decline as the frying temperature increases. This finding aligns with the work of Chaves et al. (2011), where the purified oil displayed substantially lower metal concentrations as compared to both crude oil and its corresponding biodiesel. The reduction in trace metal content in the oil and the corresponding biodiesel with increased heating suggests that the thermal stress might facilitate the vaporization of metal-containing volatiles present in the oil during the heating process. Consequently, heating or frying acts as a purification mechanism, unexpectedly leading to the production of biodiesel with reduced metal content. However, Ni exhibited an opposite trend, as it became more concentrated with increasing heating temperature due to accumulation.

The presence of trace elements and minerals in biodiesel can be attributed to the oil feedstock’s source used in the biodiesel production process. Regardless of the source, the inclusion of these elements in biodiesel can result in soap formation, insoluble deposits on automobile filters, and corrosion issues. This has been reported by various studies (Amais et al. 2012; Chaves et al. 2011; Park et al. 2019; Woods and Fryer 2007). The decline in the trace metal concentration in the oil feedstock with an increase in the heating or frying temperature presents a significant advantage for the commercialization of biodiesel, with waste frying or cooking oil gaining increasing attention for sustainability.

In contrast to biodiesel results, bioglycerol presents a reverse trend, as depicted in Fig. 5c. For instance, the concentrations of Cu, Zn, and Rb increase (e.g., from 1.1 to 2.2 ppm for Cu) with the rise in oil heating temperature. Conversely, Ni concentration decreases from 1.9 to 0.09 ppm due to the presence of high and mid-concentration elements.

Table 8 compares the trace metal levels in biodiesel produced from waste sunflower oil (Simbi et al. 2021), waste palm oil (Simbi et al. 2021), waste cooking oil (Park et al. 2019), and waste canola oil (Hossain et al. 2010) from the literature with those reported in this study. As shown in this table, trace metal levels from the literature are comparable with those found in biodiesels produced from heated canola oils at 190 and 240 °C. The slight differences in the concentration could be due to the presence of food residue in the oil and the different sources of the oil feedstock itself. Furthermore, the elemental levels reported in biodiesel are relatively similar to that of diesel with an exception of Ni. Nickel and vanadium are present in higher amounts in diesel fuel (Corbin et al. 2018) as compared to biodiesel. The lower concentration of Ni, a group I carcinogen metal, in biodiesel is an important environmental benefit that makes biodiesel a suitable alternative fuel for fossil diesel.

**Table 8:** Comparison of the concentrations of some trace metals in biodiesel from the literature and this study

Ref	Oil feedstock	Concentration in ppm						
		Zn	Cu	Sr	Ni	As	Se	Pb
Simbi et al. (2021)	Waste sunflower oil	1.624						
Simbi et al. (2021)	Waste palm oil	1.661						
This work	Heated canola oil at 240 °C	2.430	0.900	0.182	0.141	0.095	0.021	0.120
Park et al. (2019)	Waste cooking oil	2.194	0.033	0.037	0.409	0.006	0.009	
Hossain et al. (2010)	Waste canola oil (MeOH)	0.500	1.000		3.500			0.500
Corbin et al. (2018)	Diesel fuel	2.000	1.500		30.000			

The trace metal analysis of edible and inedible oil feedstocks and their corresponding biodiesel has been of great interest over the last decade due to its direct negative impact on the ecosystem. Numerous studies have been reported with respect to several oil feedstock as a part of the quality control analysis of biodiesel before commercialization (Chaves et al. 2011; Morajkar et al. 2020; Park et al. 2019), some of which were presented in Table 8. However, bioglycerol has not received considerable attention despite the overwhelming exploration of novel techniques to convert this material into value-added products such as activated carbon, carbon nanotubes, feedstock for fuel cell or for hydrogen production (Devi and Dalai 2020). Based on our results reported above (Fig. 5c), most of the metals present in the oil feedstock used for biodiesel production are carried into the bioglycerol phase. Therefore, crude bioglycerol from transesterification process would most likely require pretreatment to remove the metallic impurities depending upon its targeted application to avoid metal contamination/poisoning of the formed products or process.

## Conclusion

Response surface methodology was used to investigate biodiesel and bioglycerol production from unheated and heated canola oils. The optimal conditions for biodiesel and bioglycerol production from oils were, respectively, methanol/oil ratio of 12:1 and 4.25:1, catalyst wt% of 1.0 and 2.93wt%, and reaction time of 60 and 119.15 min. With increasing oil temperature, the trace metal concentration reduced in biodiesel and increased in biogylcerol. This indicates the suitability of heated/waste oil in producing biodiesel with low metal content. The increased metal concentration in bioglycerol from heated oil can limit its applications in catalytic processes such as fuel cells, material synthesis, and H_2_ production.

### Supplementary Information

Below is the link to the electronic supplementary material.

Supplementary file1 (PDF 1562 KB)

## Data Availability

All data generated or analyzed during this study are included in this published article and its supplementary information files.
