# Correction to: An ICP‑MS study on metal content in biodiesel and bioglycerol produced from heated and unheated canola oils

**DOI:** 10.1007/s11356-023-31334-6

**Published:** 2023-12-04

**Authors:** Rukayat S. Bojesomo, Abhijeet Raj, Mirella Elkadi, Mohamed I. Hassan Ali, Sasi Stephen

**Affiliations:** 1 Department of Chemistry, Khalifa University of Science and Technology, P.O Box: 127788, Abu Dhabi, United Arab Emirates; 2 Department of Chemical Engineering, Indian Institute of Technology Delhi, New Delhi, 110016 India; 3 Department of Mechanical Engineering, Khalifa University of Science and Technology, P.O Box: 127788, Abu Dhabi, United Arab Emirates

**Correction to: Environmental Science and Pollution Research (2023) 30:115064–115080**

10.1007/s11356-023-30004-x

The article "An ICP‑MS study on metal content in biodiesel and bioglycerol produced from heated and unheated canola oils" written by Rukayat S. Bojesomo, Abhijeet Raj, Mirella Elkadi, Mohamed I. Hassan Ali and Sasi Stephen was originally published electronically on the publisher’s internet portal on 25 October 2023 without open access. With the author(s)’ decision to opt for Open Choice the copyright of the article changed on 26 November 2023 to © The Author(s) 2023 and the article is forthwith distributed under a Creative Commons Attribution 4.0 International License, which permits use, sharing, adaptation, distribution and reproduction in any medium or format, as long as you give appropriate credit to the original author(s) and the source, provide a link to the Creative Commons licence, and indicate if changes were made. The images or other third-party material in this article are included in the article’s Creative Commons licence, unless indicated otherwise in a credit line to the material. If material is not included in the article’s Creative Commons licence and your intended use is not permitted by statutory regulation or exceeds the permitted use, you will need to obtain permission directly from the copyright holder. To view a copy of this licence, visit http://creativecommons.org/licenses/by/4.0.

The Original article has been corrected.

