# Supplementary material for: An ICP-MS study on metal content in biodiesel and bioglycerol produced from heated and unheated canola oils
Source: Environ Sci Pollut Res Int. 2023 Oct 25;30(54):115064–80. doi: 10.1007/s11356-023-30004-x (PMC10691977; doi:10.1007/s11356-023-30004-x)
Supplement: Supplementary file 1 — Supplementary file1 (PDF 1562 KB) [file 11356_2023_30004_MOESM1_ESM.pdf]

SUPPORTING INFORMATION

An ICP-MS Study on Metal Content in Biodiesel and Bioglycerol Produced from Heated and Unheated Canola Oils

Rukayat S. Bojesomo[1], Abhijeet Raj[2,3,], Mirella Elkadi[1,*], Mohamed I. Ali[4,*], Sasi Stephen[1]*

[1]Department of Chemistry, Khalifa University of Science and Technology, P.O Box: 127788, Abu Dhabi, United Arab Emirates

[2]Department of Chemical Engineering, Khalifa University of Science and Technology, P.O Box: 127788, Abu Dhabi, United Arab Emirates

[3]Centre for Catalysis and Separation, Khalifa University of Science and Technology, P.O Box: 127788, Abu Dhabi, United Arab Emirates

[4]Department of Mechanical Engineering, Khalifa University of Science and Technology, P.O Box: 127788, Abu Dhabi, United Arab Emirates

Email: 100059967@ku.ac.ae (R.B.)

Content

Statistical analysis: fit summary -- 3

Diagnostic test --- 3-4

Conventional single factor optimization of biodiesel production --- 4-5

Optimization of transesterification reaction parameters for crude bioglycerol production ----------------- 6-14

Reference --- 14

Statistical analysis: fit summary

Tables S1 present the summary of all the models analyzed using DesignExpert software. The quadratic model is the most suitable with the highest R^2 value, and has therefore been employed for RSM analysis of biodiesel production.

Table S1 | Model summary statistics for BD_100, BD_190, and BD_240

Source	BD_100					BD_190					BD_240				
	Sequential p-value	Lack of Fit p-value	Adjusted R^2	Predicted R^2		Sequential p-value	Lack of Fit p-value	Adjusted R^2	Predicted R^2		Sequential p-value	Lack of Fit p-value	Adjusted R^2	Predicted R^2	
Linear	< 0.0001	< 0.0001	0.6935	0.4840		**< 0.0001**	**< 0.0001**	**0.7678**	**0.5391**	**Suggested**	**0.0478**	**0.5009**	**0.2657**	**0.0684**	**Suggested**
2FI	0.8638	< 0.0001	0.6430	0.4004		0.7289	< 0.0001	0.7405	0.2734		0.3522	0.5119	0.2909	-0.4465	
**Quadratic**	**< 0.0001**	**0.0062**	**0.9698**	**0.8763**	**Suggested**	**0.0172**	**0.0001**	**0.8726**	**0.1561**	**Suggested**	0.7643	0.3692	0.1742	-1.4171	
Cubic	0.0062		0.9958		**Aliased**	0.0001		0.9963		**Aliased**	0.3692		0.3031		**Aliased**

Diagnostic Test

Diagnostic testing was carried out using DesignExpert software to validate the model for its accuracy by analyzing the normal plot for biodiesel (Figure S1) and residual vs run number for the produced biodiesel (Figure S2) for different heating temperatures of the canola oil feedstock utilized for production.

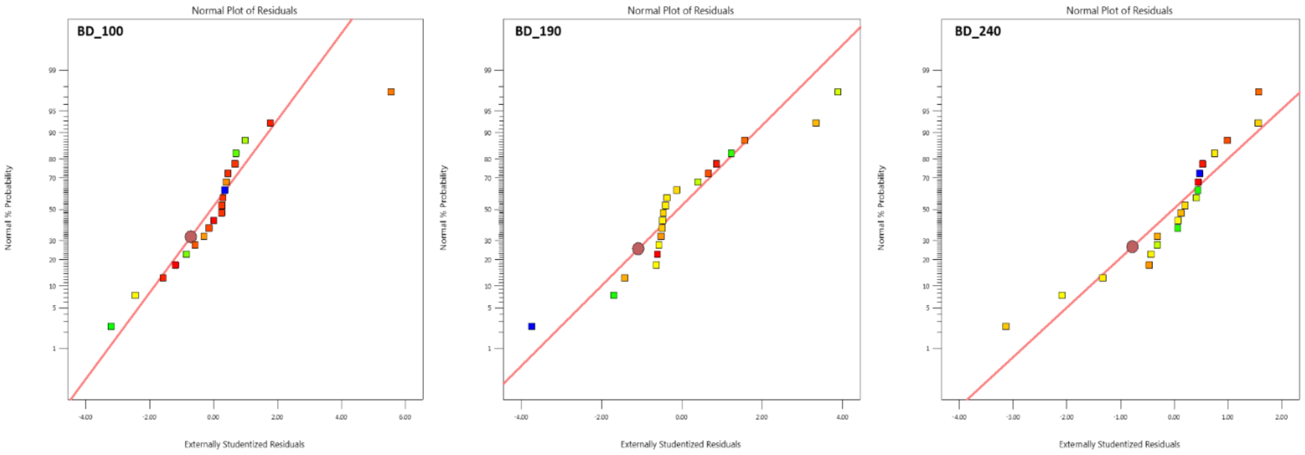

Figure S1 | Normal plot of residual for the response BD_100, BD_190, and BD_240

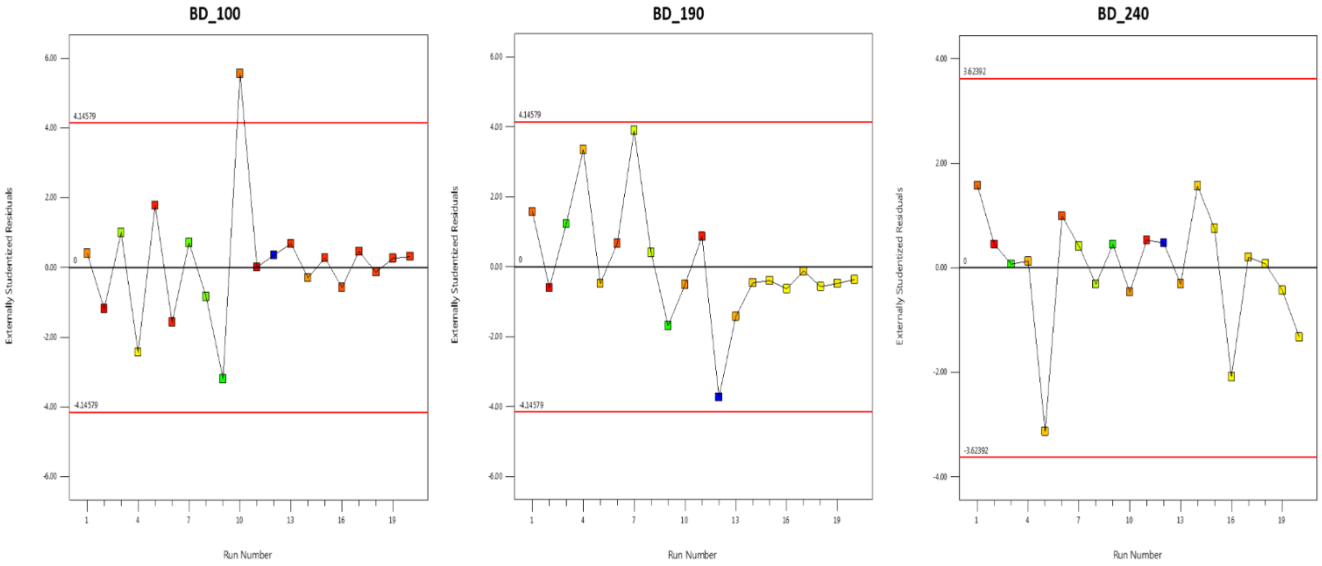

Figure S2 | Residual vs run plot BD_100, BD_190, and BD_240

Conventional single factor optimization of biodiesel production

Conventional single factor optimization was carried out as a comparative analysis to the CCD-based RSM analysis for optimization of biodiesel from both unheated (dried at 100 ^{o}C to remove moisture content) and heated (heated at 190 ^{o}C and 240 ^{o}C) canola oils. The effect of (1) Methanol/Oil molar ratio with a constant 1 wt% KOH and a reaction time of 60 minutes (Figure S3a), (2) catalyst concentration with a constant Methanol/Oil molar ratio of 12:1 and a reaction time of 60 minutes (Figure S3b), and (3) reaction time with a constant Methanol/Oil molar ratio of 12:1 (Figure S3c) on biodiesel yield are presented.

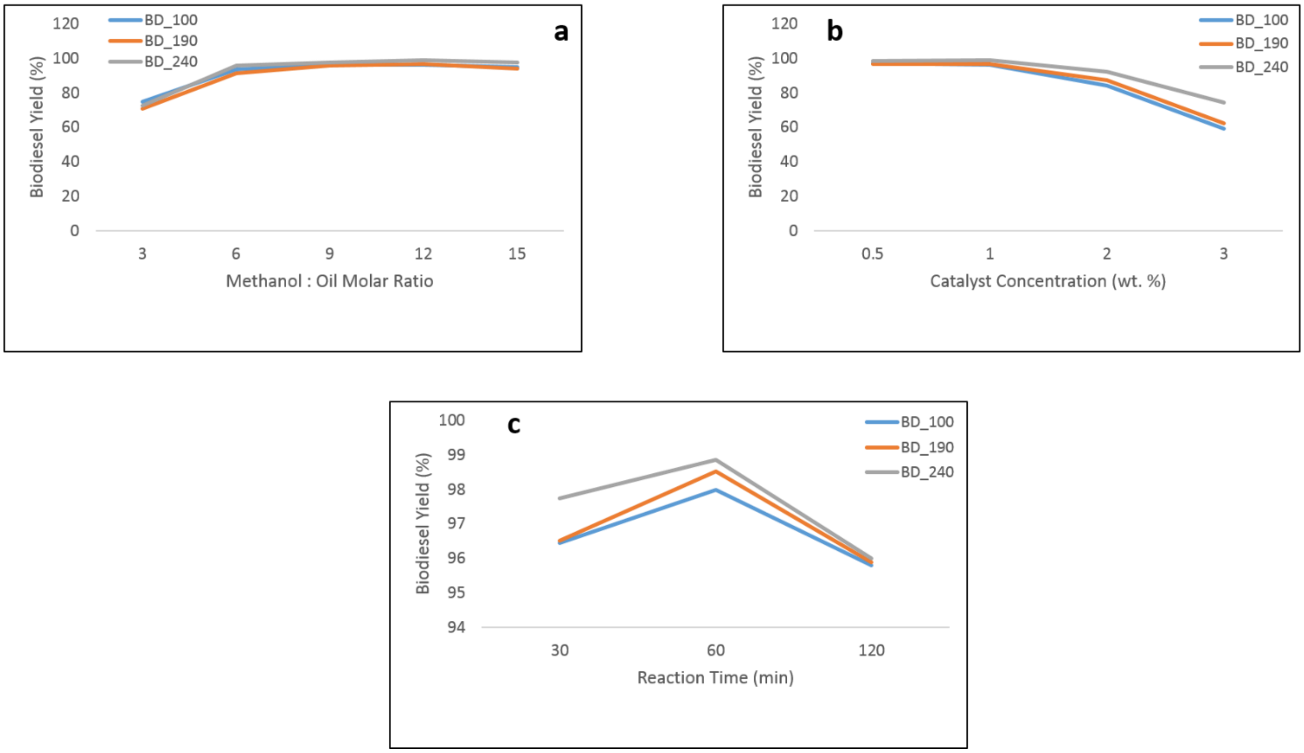

Figure S3 | Single-factor optimization plots on the effect of (a) Methanol/Oil molar ratio, (b) catalyst concentration, and (c) reaction time on the yields of biodiesels from unheated canola oil (BD_100), from heated canola oil at 190 ^{o}C (BD_190), and from heated canola oil at 240 ^{o}C (BD_240).

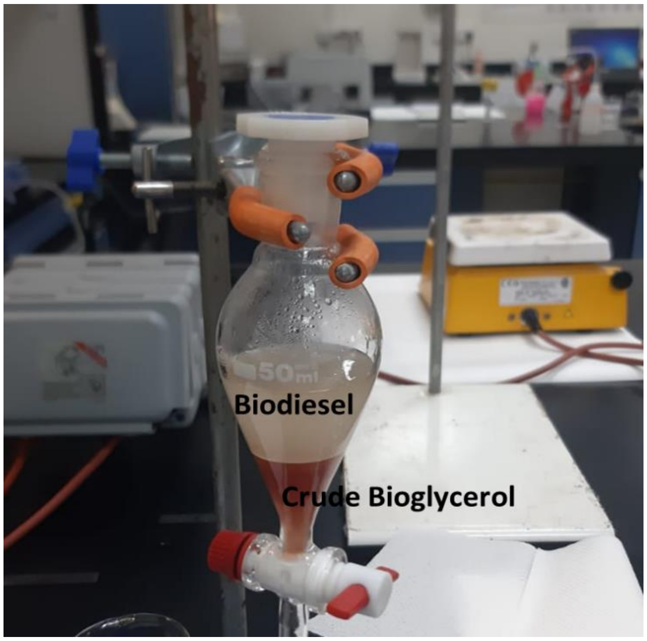

Figure S4 | Photograph of the separation phase of biodiesel and Bioglycerol.

Optimization of transesterification reaction parameters for crude bioglycerol production

The production of crude bioglycerol (BG) was carried out in a single-step homogenous catalyzed transesterification process. The optimization of independent variables, *i.e.*, M/O ratio, catalyst wt. % (wt. %), and reaction time, were studied. Conventional single factor optimization and RSM in combination with CCD were employed to determine the interaction between independent operational variables studied.

Table S2 | Full factorial central composite design matrix for bioglycerol production

Run	Independent Variables			Response (BG_100)		Response (BG_190)		Response (BG_240)	
	A: Methanol: Oil Ratio	B: Catalyst concentration (wt. %)	C: Reaction Time (min)	Actual Yield (%)	Predicted Yield (%)	Actual Yield (%)	Predicted Yield (%)	Actual Yield (%)	Predicted Yield (%)
1	-1 (6.00)	-1 (1.00)	-1 (60.00)	10.37	11.19	12.54	12.42	10.59	10.49
2	+1 (12.00)	-1 (1.00)	-1(60.00)	10.64	9.09	11.00	8.00	10.39	10.98
3	-1 (6.000)	+1 (2.00)	-1 (60.00)	18.25	20.47	26.94	25.45	12.83	13.10
4	+1 (12.00)	+1 (2.00)	-1 (60.00)	15.89	16.92	13.56	15.52	14.40	14.77
5	-1 (6.00)	-1 (1.00)	+1 (100.00)	14.622	14.03	14.90	12.96	11.23	10.75
6	+1 (12.00)	-1 (1.00)	+1 (100.00)	12.34	10.56	11.86	13.37	10.15	9.78
7	-1 (6.00)	+1 (2.00)	+1 (100.00)	23.85	25.84	20.56	23.58	15.40	14.70
8	+1 (12.00)	+1 (2.00)	+1 (100.00)	21.31	20.92	18.34	18.48	14.93	14.93
9	-α (3.00)	0 (1.50)	0 (80.00)	24.02	22.02	24.56	24.84	11.99	12.44
10	+α (15.00)	0 (1.50)	0 (80.00)	13.44	15.00	15.62	15.32	13.50	13.15
11	0 (9.00)	-α (0.50)	0 (80.00)	7.77	9.98	9.73	11.87	9.23	9.36
12	0 (9.00)	+α (3.00)	0 (80.00)	45.35	44.17	48.10	47.14	21.62	21.61
13	0 (9.00)	0 (1.50)	-α (30.00)	11.77	11.03	11.06	12.01	13.50	13.07
14	0 (9.00)	0 (1.50)	+α (120.00)	16.85	17.56	16.24	14.74	11.96	12.75
15	0 (9.00)	0 (1.50)	0 (80.00)	11.66	12.07	9.64	10.87	10.46	11.17
16	0 (9.00)	0 (1.50)	0 (80.00)	13.54	12.07	11.68	10.87	9.42	11.17
17	0 (9.00)	0 (1.50)	0 (80.00)	12.55	12.07	11.68	10.87	12.93	11.17
18	0 (9.00)	0 (1.50)	0 (80.00)	12.65	12.07	10.58	10.87	11.95	11.17
19	0 (9.00)	0 (1.50)	0 (80.00)	11.99	12.07	10.99	10.87	11.54	11.17
20	0 (9.00)	0 (1.50)	0 (80.00)	12.35	12.07	11.34	10.87	10.90	11.17

i. Statistical Analysis

The predicted and actual bioglycerol yields are presented in Table S2. An analysis of variance (ANOVA) was employed to determine the significance and fitness of the quadratic regression model. Table SS3 provides the ANOVA summary for the full quadratic model for the percentage yield of bioglycerol from unheated (CO_100) and heated canola oils (CO_190 and CO_240).

Table S3 | ANOVA summary for the full quadratic model for percentage yield of biodiesel from unheated and heated canola oil

Source		BG_100					BG_190					BG_240				
	df	Sum of Squares	Mean Square	F-value	p-value		Sum of Squares	Mean Square	F-value	p-value		Sum of Squares	Mean Square	F-value	p-value	
**Model**	**9**	**1232.86**	**136.98**	**40.98**	**< 0.0001**	**significant**	**1486.52**	**165.17**	**39.21**	**< 0.0001**	**significant**	**137.22**	**15.25**	**15.15**	**0.0001**	**significant**
A-Methanol:Oil Molar Ratio	1	33.71	33.71	10.08	0.0099		111.88	111.88	26.56	0.0004		1.71	1.71	1.70	0.2212	
B-Catalyst Concentration	1	795.09	795.09	237.84	< 0.0001		924.38	924.38	219.42	< 0.0001		99.71	99.71	99.06	< 0.0001	
C-Reaction Time	1	36.14	36.14	10.81	0.0082		1.08	1.08	0.2561	0.6238		0.1142	0.1142	0.1134	0.7432	
AB	1	1.05	1.05	0.3136	0.5878		15.20	15.20	3.61	0.0867		0.7021	0.7021	0.6975	0.4231	
AC	1	0.9385	0.9385	0.2807	0.6078		11.67	11.67	2.77	0.1271		1.05	1.05	1.04	0.3309	
BC	1	3.20	3.20	0.9574	0.3509		2.91	2.91	0.6896	0.4257		0.9153	0.9153	0.9094	0.3628	
A^2	1	64.25	64.25	19.22	0.0014		131.33	131.33	31.17	0.0002		4.08	4.08	4.06	0.0717	
B^2	1	229.71	229.71	68.72	< 0.0001		390.73	390.73	92.75	< 0.0001		16.33	16.33	16.22	0.0024	
C^2	1	10.68	10.68	3.20	0.1041		11.20	11.20	2.66	0.1341		4.70	4.70	4.66	0.0561	
Residual	10	33.43	3.34				42.13	4.21				10.07	1.01			
**Lack of Fit**	**5**	**31.34**	**6.27**	**14.99**	**0.0050**	**significant**	**39.05**	**7.81**	**12.70**	**0.0072**	**significant**	**2.58**	**0.5157**	**0.3444**	**0.8665**	**not significant**
Pure Error	5	2.09	0.4180				3.08	0.6151				7.49	1.50			
Cor Total	19	1266.29					1528.64					147.29				

The model F-values of 40.98, 39.21, and 15.15 suggest that the model is significant and there is just a 0.01% opportunity that an F-value this huge could happen because of noise for BG_100, BG_190, and BG_240, respectively (Table SS3). The P-values of under 0.05 shows that the model terms, A, B, C, A^2, and B^2 are noteworthy. The lack of fit F-values of 14.99 and 12.70 for BG_100 and BG_190, respectively, suggest that the absence of fit is significant, while its value of 0.34 for BG_240 infers that the lack of fit isn't huge as compared to its pure error. The predicted R^2 is in sensible agreement with the adjusted R^2, i.e., the difference is below 0.2 for BG_100, BG_190, and BG_240. The adequate precision measures the signal-to-noise ratio. A ratio of more than 4 is desirable. The ratios of 27.131, 26.963, and 17.270 for BG_100, BG_190, and BG_240, individually, show a satisfactory signal, and this model can be utilized to explore the design space. Eqns. 1, 2, and 3 show the adjusted model coding conditions for unrefined or crude bioglycerol yields comparable to the significant variables.

$$Y_{BG\_100} = 14.53 - 3.70A + 16.70B + 3.98C - 1.81AB - 1.54AC + 3.56BC + 6.44A^2 + 12.07B^2 + 2.62C^2 \quad (1)$$

$$Y_{BG\_190} = 13.66 - 6.74A + 18.01B + 0.69C - 6.89AB + 5.43AC - 3.39BC + 9.21A^2 + 15.74B^2 + 2.69C^2 \quad (2)$$

$$Y_{BG\_240} = 12.22 + 0.83A + 5.91B + 0.22C + 1.48AB - 1.63AC + 1.90BC + 1.62A^2 + 3.221B^2 + 1.74C^2 \quad (3)$$

Where Y_{BG_100}, $Y_{BG\_190,}$ and Y_{BG_240} are the predicted percentage yields of crude bioglycerol from unheated canola oil (dried at 100 ^oC), heated canola oil at 190^oC, and heated canola oil at 240 ^oC respectively, A is the M/O ratio, B is the catalyst wt. % (wt. %), and C is the reaction time (min).

The significance of each parameter included in this study to the bioglycerol yield was considered and evaluated by the probability value (p-value), as listed in Table S3. A p-value of less than 0.005 indicates a significant effect of those parameters at a 95% confidence level. As observed in the results of the variance analysis (ANOVA) (Table SS3), the catalyst wt. % is the most significant parameter, with a p-value < 0.005 for all the oil conditions. The M/O ratio is the second significant factor for BG_100 and BG_190 and less significant for BG_240. This is similar to the results obtained by Silva et al. [1], where the alcohol ratio was found to be significant for bioglycerol production from soybean oil with ethanol. However, reaction time is the least significant parameter of all the responses studied, as indicated by a p-value above 0.05 and a lower F-value compared to the other parameters.

Table S4 | Model summary statistics for BG_100, BG_190, and BG_240.

Source	BG_100					BG_190					BG_240				
	Sequentia l p-value	Lack of Fit p-value	Adjusted R^2	Predicted R^2		Sequentia l p-value	Lack of Fit p-value	Adjusted R^2	Predicted R^2		Sequentia l p-value	Lack of Fit p-value	Adjusted R^2	Predicted R^2	
Linear	< 0.0001	0.0001	0.7272	0.4910		0.0005	< 0.0001	0.5967	0.2793		< 0.0001	0.3569	0.7464	0.6277	
2FI	0.9704	< 0.0001	0.6703	0.2846		0.8504	< 0.0001	0.5320	0.0427		0.7541	0.2728	0.7144	0.5407	
**Quadratic**	**< 0.0001**	**0.0050**	**0.9498**	**0.7133**	**Suggested**	**< 0.0001**	**0.0072**	**0.9476**	**0.7606**	**Suggested**	**0.0119**	**0.8665**	**0.8702**	**0.7743**	**Suggested**
Cubic	0.0050		0.9937		**Aliased**	0.0072		0.9924		**Aliased**	0.8665		0.8068		**Aliased**

In general, the model (quadratic) is satisfactory (Table S4) for exploring the experimental relationship between the variable and the response (yield) within the range of the experimental variable studied. As shown in Table S4, the quadratic model has a maximum adjusted R^2 value of 0.9498, 0.9476, and

0.8702 for BG_100, BG_190, and BG_240, respectively, and the difference between the adjusted R^2 value and the predicted R^2 value is < 0.2. The coefficient of determination R^2 signifies approximately 95% of the variability in the response could be explained by the model for BG_100 and BG_190, and 87% for BG_240, respectively.

ii. Diagnostic test (affirming the numerical model)

The model was validated by testing its accuracy by analyzing the residual and leverage (**Error! Reference source not found.** in the manuscript). Table S2 shows the predicted response values and the actual experimental response values for BG_100, BG_190, and BG_240. It can be observed that the developed model adequately describes the experimental range studied, and the observations with high leverage have a strong influence on the coefficients in the regression model. As listed in Table S2, the actual yield is close to the predicted yield.

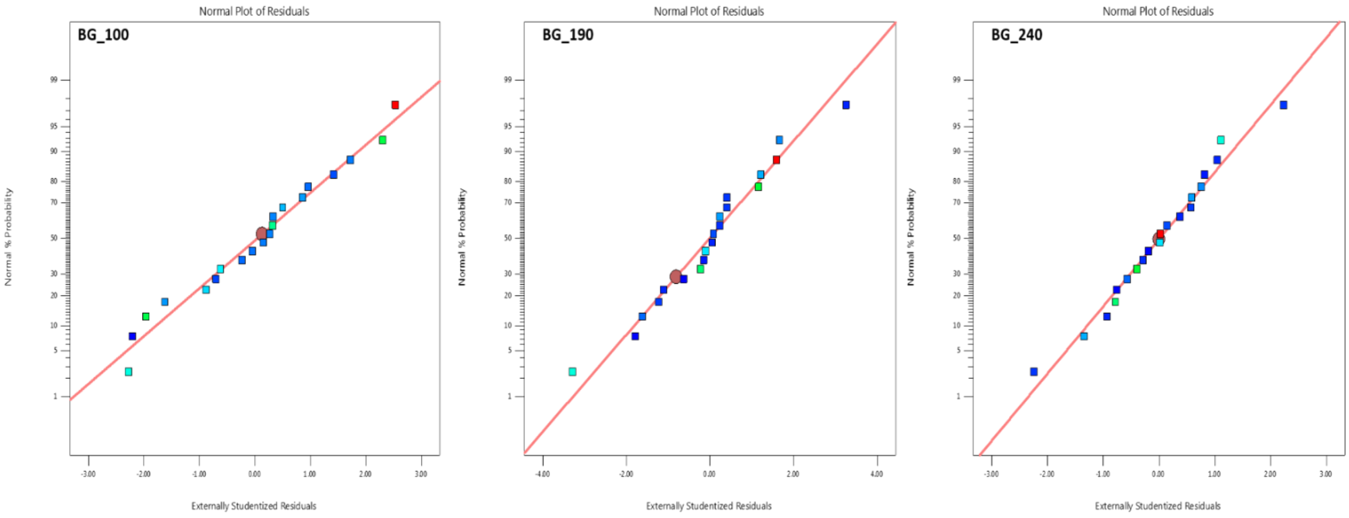

Figure S5 | Normal plot of residuals for the response for BG_100, BG_190, and BG_240.

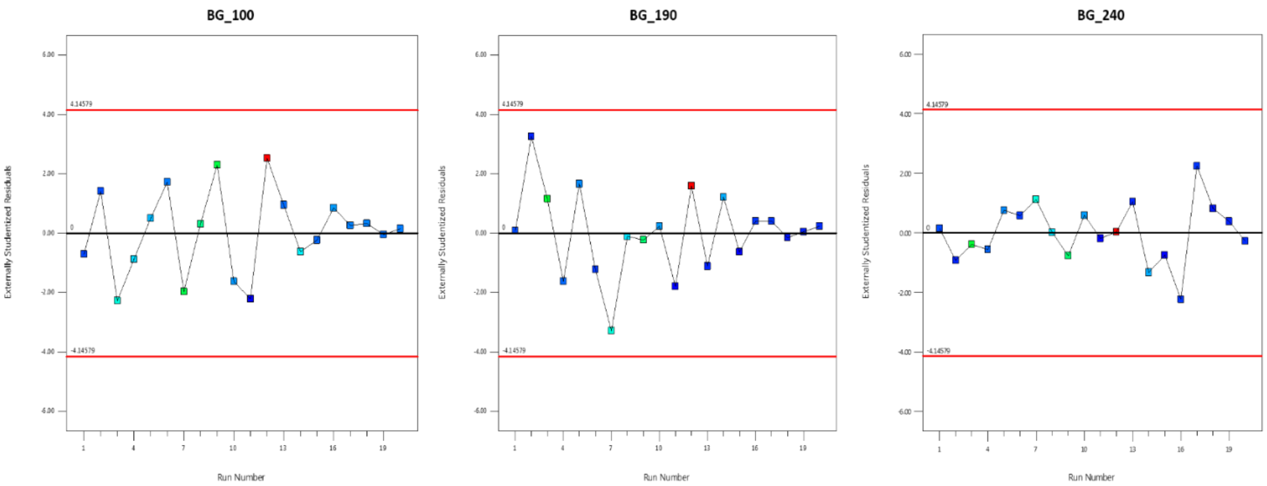

Figure S6 | Residual vs run plots for BG_100, BG_190, and BG_240.

Figure S5 shows a normal plot, illustrating how the data points are scattered randomly. It can be observed that more than 85% of the points are aligned on the standard line, which validates the fitness of the model for all the responses. Figure S6 shows the residual plot against the run order. The random pattern of the residual also signifies model accuracy.

iii. The interactions of the independent factors

Figure S7 indicates the contour plots of the crude bioglycerol yield (%) for heated and unheated canola oil. As shown in Figure S7, there is an interaction between all the independent variables studied. Therefore, all of the parameters cannot be analyzed independently for BG_100, BG_190, and BG_240. For each contour plot, two independent variables (A, B, and C) are plotted while the third variable is kept constant at a medium value (M/O ratio of 9:1, catalyst wt. % of 1.5 wt.%, and reaction time of 90 minutes). These plots demonstrate the dependence and change in response value, as the experimental conditions are altered. The interaction effects of the parameters, M/O ratio-catalyst wt. % (MC), M/O ratio-reaction time (MR), and catalyst wt. %-reaction time (CR), are considered for all the responses (BG_100, BG_190, and BG_240). A slightly weaker interaction is observed between CR, and this could be associated with the less significant reaction time, as indicated by the ANOVA results (Table SS3).

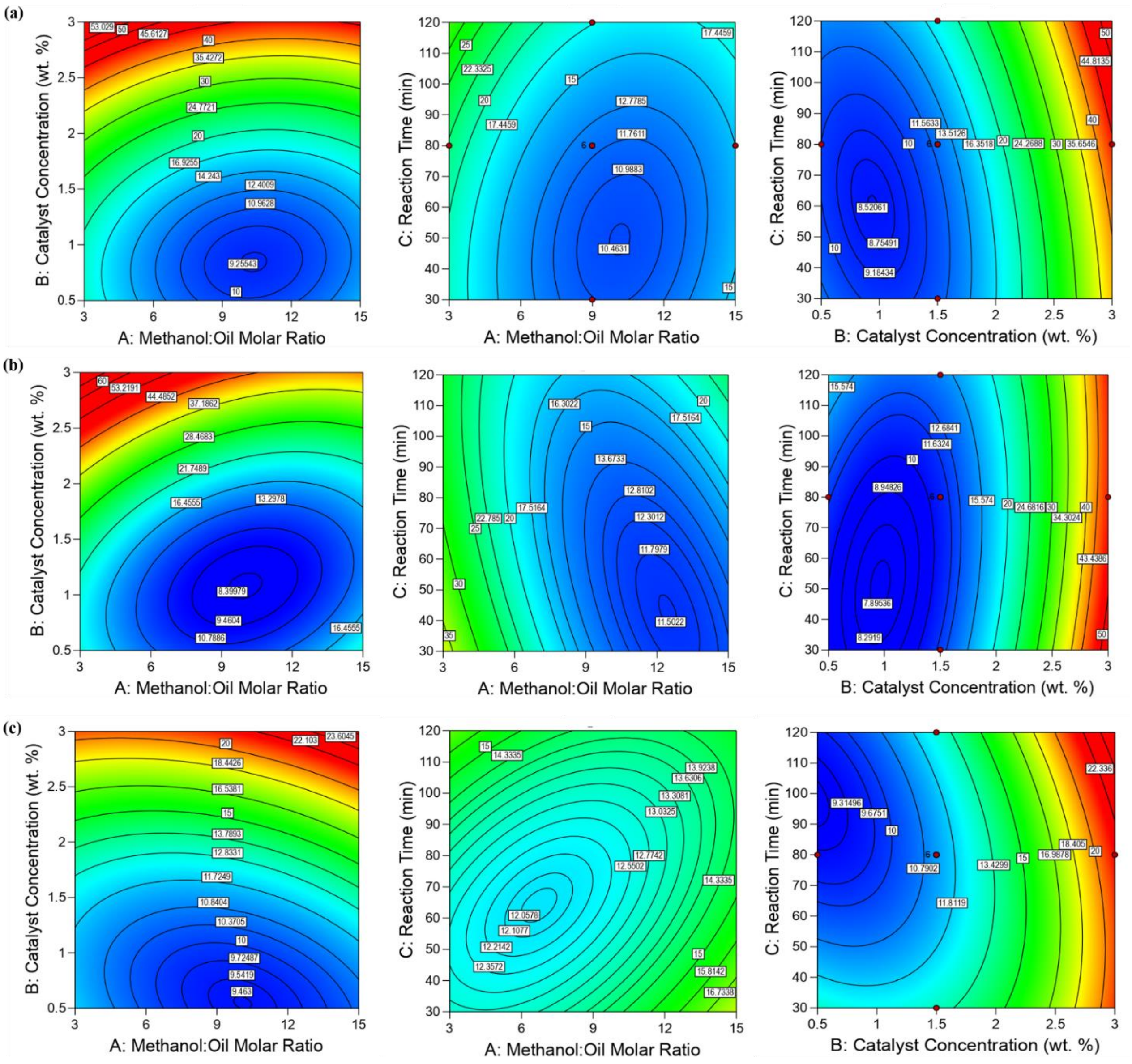

Figure S7 | Contour plots on interaction effect of M/O ratio and catalyst wt. % (left), M/O ratio and reaction time (middle), and catalyst wt. % and reaction time (right) on bioglycerol yield from: (a) unheated canola oil (BG_100); (b) heated canola oil at 190 °C (BG_190); and (c) heated canola oil at 240 °C (BG_240).

Effects of operating parameters on crude bioglycerol yield

The effects of the process variables, M/O ratio (A), catalyst wt% (B), and reaction time (C) on the percentage yield of crude bioglycerol were analyzed by plotting contour plots of any two independent variables with the third variable constant at the medium value. The contour plots with respect to the effects of the independent variable are shown in Figures S7a–S7c for the RSM analysis and Figure S8a–S8c for the conventional single factor optimization analysis.

A. Effects of M/O ratio

Figure S7 shows the effect of Methanol/Oil ratio, catalyst wt%, and reaction time (right) on bioglycerol yield from (a) unheated oil bioglycerol (BG_100); (b) heated oil at 190 ^{o}C bioglycerol (BG_190); and (c) heated oil at 240 ^{o}C bioglycerol (BG_240). It shows the impact of two variables while keeping the third variable fixed at a medium value. According to Figure S7 for the RSM analysis and Figure S8 for the convention single factor optimization, there is a decrease in the yield of crude bioglycerol with an increasing M/O ratio from 3:1 to 12:1, with a slight increase observed above the 12:1 molar ratio. These results complement the previous results showing that the biodiesel yield increased with increasing M/O ratio from 3:1 to 12:1 and decreased above 12:1 due to excess alcohol leading to recombination of esters and glycerol to monoglyceride [2]. As a result, more crude bioglycerol is produced at a low molar ratio of methanol for both the unheated and heated oil included in this study. High catalyst wt% (above 2.5 wt. %) with a M/O ratio between 3:1 and 9:1 is enough to produce over 25% yield of crude bioglycerol for canola oil heated at 100 ^{o}C and 190 ^{o}C for two hours, respectively. However, in the case of canola oil heated at 240 ^{o}C for two hours, a higher Methanol/Oil molar ratio is required. In general, the yield of crude bioglycerol decreases with increasing frying temperature of the oil feedstock. This is due to the increase in the free fatty acid (FFA) content, which leads to soap formation as compared to those fried at a lower temperature [3]. This might be the apparent cause of the decrease in the yield of crude bioglycerol.

B. Effects of catalyst concentration

Figure S7a-S7c depict the effects of catalyst wt. % and M/O ratio (left) and catalyst wt. % and reaction time (right) while keeping the third variable in the medium range. The effects of catalyst weight percent were studied in the range of 0.5 - 3.0 wt% for both RSM and conventional single factor optimization analysis. The yield of crude bioglycerol continuously increased with increasing catalyst wt%, indicating the positive impacts of this parameter on the bioglycerol yield, which complement the ANOVA results, indicating that the catalyst wt% is the most significant variable. Similar trends were observed from the convention single factor analysis (Figure S8). The maximum yield of 34–48% was achieved at 3.0 wt. % of catalyst loading in both the RSM and single-factor analysis for all the bioglycerol produced for all the oil conditions studied. Excess catalyst wt% causes saponification (soap formation), which leads to emulsification of esters and glycerol and results in biodiesel loss [2, 4]. On the other hand, this favors the yield of the crude bioglycerol in terms of weight percent, although it might not have significant effects on the glycerol content of the crude bioglycerol produced. As a result, high catalyst wt. % (3.0 wt. %) coupled with a high methanol molar ratio and reaction positively influences the yield of the crude bioglycerol phase.

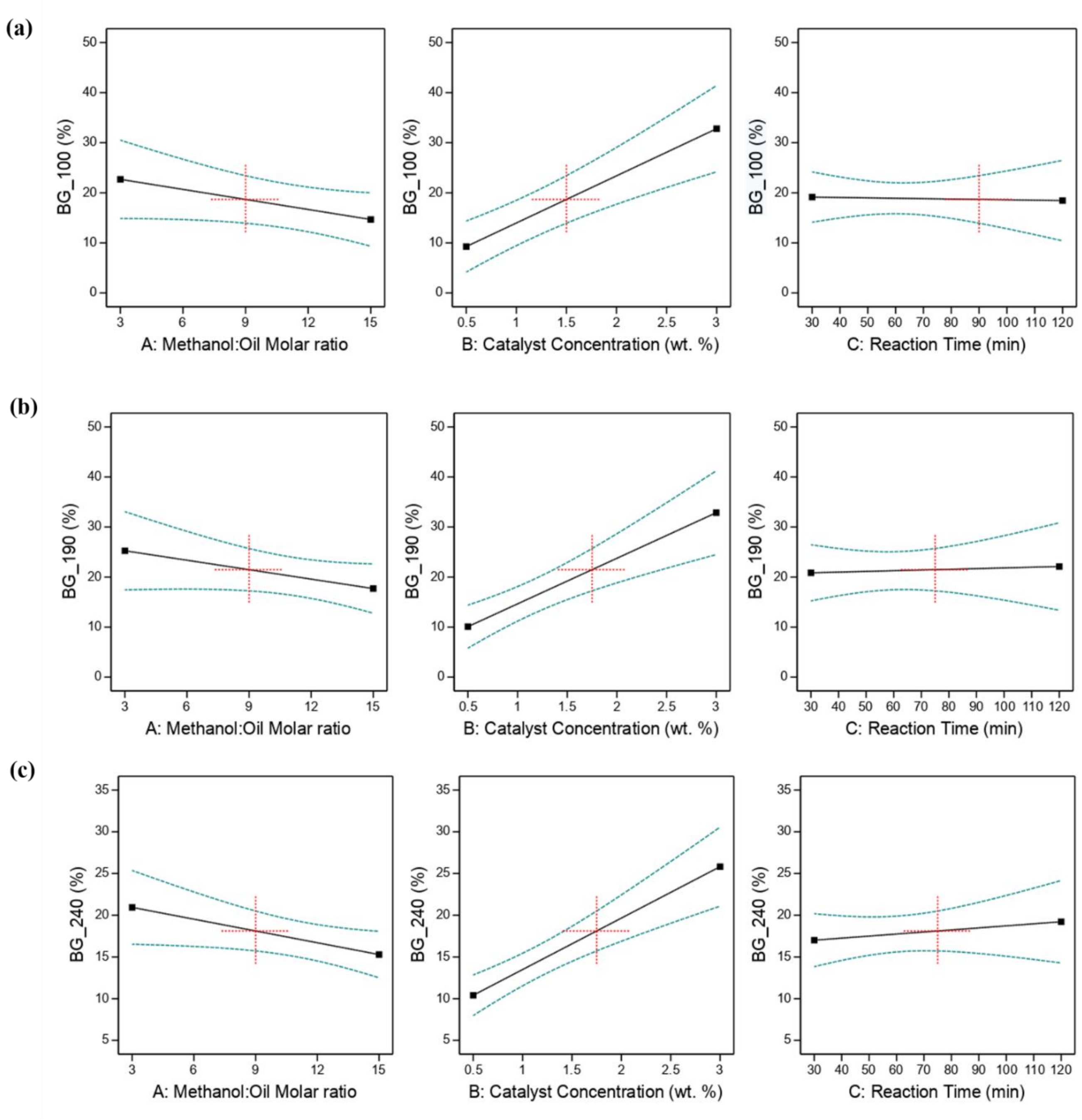

Figure S8 | Conventional Single factor plots on the effect of (a) Methanol/Oil ratio (left), catalyst wt. % (middle), and reaction time (right) on the yield of bioglycerol produced from (a) unheated canola oil (BG_100); (b) heated canola oil at 190 °C (BG_190); and (c) heated canola oil at 240 °C (BG_240).

C. Effects of reaction time

Reaction time is the least significant parameter for both biodiesel and bioglycerol yields. However, its influence is slightly more significant on crude bioglycerol compared to biodiesel within the range

included in this study. Transesterification reactions were carried out at a constant speed between 30 and 120 minutes of reaction time in this study. Figure S7 (a-c) and Figure S8 (a-c) (middle and right) show the effects of reaction time and M/O ratio and reaction time with catalyst wt% on the yield of crude bioglycerol for RSM analysis and conventional single factor optimization analysis. From Figure S7, it can be seen that the contour plots in relation to the reaction time show no significant effects on the response.

Similarly, the single factor plots (Figure S8) show a comparatively horizontal line. This signifies the lesser significance of this parameter to the response, which is in agreement with the ANOVA results. With an increased reaction time, accompanied by high catalyst wt. %, there is a slight gradual increase in the yield of the crude bioglycerol. The more prolonged reaction can enable the completion of the transesterification reaction on a positive note and enhance the yield of biodiesel. However, the possibility of the reverse reaction - recombination of ester and glycerol to monoglyceride- in the presence of a high molar ratio of alcohol and the saponification reaction in the presence of a high catalyst wt% cannot be neglected. These two reactions can be favored with a longer reaction time and lead to loss of biodiesel, but they can also positively influence the yield of the crude bioglycerol due to accumulation of either glycerol content or other waste content such as soap in the glycerol phase. This slight increase in the crude bioglycerol yield with increasing reaction time complements the decrease in the biodiesel yield above 60 minutes of reaction time observed.

References

1. Silva, G.F., F.L. Camargo, and A.L.O. Ferreira, *Application of response surface methodology for optimization of biodiesel production by transesterification of soybean oil with ethanol.* Fuel Processing Technology, 2011. **92**(3): p. 407-413.

2. Harabi, M., et al., *Biodiesel and Crude Glycerol from Waste Frying Oil: Production, Characterization and Evaluation of Biodiesel Oxidative Stability with Diesel Blends.* Sustainability, 2019. **11**(7): p. 1937.

3. Giuffrè, A.M., C. Zappia, and M. Capocasale, *Effects of High Temperatures and Duration of Heating on Olive Oil Properties for Food Use and Biodiesel Production.* Journal of the American Oil Chemists' Society, 2017. **94**(6): p. 819-830.

4. Atapour, M., H.-R. Kariminia, and P.M. Moslehabadi, *Optimization of biodiesel production by alkali-catalyzed transesterification of used frying oil.* Process Safety and Environmental Protection, 2014. **92**(2): p. 179-185.
